# A novel resistance locus from an unexploited East Asian *Vitis coignetiae* confers resistance to grapevine downy mildew

**DOI:** 10.1007/s00122-026-05273-y

**Published:** 2026-06-01

**Authors:** Nagarjun Malagol, Anna Werner, Reinhard Töpfer, Ludger Hausmann

**Affiliations:** Julius Kühn Institute (JKI), Federal Research Centre for Cultivated Plants, Institute for Grapevine Breeding, Geilweilerhof, 76833 Siebeldingen, Germany

## Abstract

**Key message:**

A novel locus for grapevine resistance to *Plasmopara viticola* (*Rpv32*) was identified on chromosome 14 in the East Asian wild species *Vitis coignetiae* and compared with other resistances.

**Abstract:**

One of the most devastating diseases in viticulture worldwide is downy mildew, caused by an obligate biotrophic oomycete, *Plasmopara viticola*. While fungicides are widely used to prevent yield losses, their environmental impact and decreasing social acceptance highlight the need for sustainable alternatives. Breeding and cultivating resistant grapevine varieties is an effective option for durable disease control, but requires the identification of novel genetic resistances. To minimize disease impact and pathogen adaptation, diverse genetic resistance mechanisms are essential. We conducted a QTL analysis to identify a new resistance locus in the unexploited East Asian wild species *Vitis coignetiae*. A biparental F1 population derived from ‘Morio Muskat’ × COxGT2 (*V. coignetiae* × ‘Gewürztraminer’) was evaluated using artificial infection assays over three consecutive years and genotyped with rhAmpSeq markers. A high-resolution genetic map comprising 639 markers spanned 1147.36 cM across 19 linkage groups, covering 96% of the physical genome. QTL mapping identified a highly significant and stable QTL on chromosome 14 in three years that explains up to 28.16% of the phenotypic variation. This novel resistance locus was named *Rpv32*. Microscopic analysis revealed restricted intracellular pathogen development in the resistant parent, and pathogen proliferation assays confirmed the strength of *Rpv32*-mediated resistance. An additional QTL for leaf hair density was detected on chromosome 5, but this trait does not appear to play a role in downy mildew resistance in this population. Markers tightly linked to *Rpv32* have been developed, enabling the rapid utilization of this novel resistance in breeding through introgression and pyramiding using marker-assisted selection.

**Supplementary Information:**

The online version contains supplementary material available at 10.1007/s00122-026-05273-y.

## Introduction

Globally, grapevines belong to the economically most important perennial fruit crops. *Vitis vinifera L.* is the predominantly cultivated grapevine species yet highly vulnerable to numerous invasive fungal pathogens (Koledenkova et al. 2022). The catastrophic effects of these fungal pathogens on yield can be mainly seen during the pre-and post-harvest time (Armijo et al. 2016). Among several pathogens, downy mildew (DM) is one of the most destructive grapevine diseases, caused by the obligate biotrophic oomycete, *Plasmopara viticola* (Berk. & Curt.) Berl. & de Toni. The polycyclic pathogen *P. viticola*, which originates from North America, is able to attack all the green tissues of the plant (leaves, shoot tips, inflorescences and young berries) under optimal conditions by invading the stomata (Kassemeyer 2015). Characteristic early symptoms of DM on the leaf include yellow oil spots on the adaxial leaf surface, and at the later stage, white cottony growth occurs on the abaxial leaf surface. DM results in severe damage to leaves up to rapid defoliation and affects fruit development, composition, and ultimately yield and quality (Gessler et al. 2011; Scott et al. 2022). Serious yield losses caused by DM have been reported mainly in temperate and tropical grape growing regions of the world (Marguerit et al. 2009; Koledenkova et al. 2022).

Currently, a convenient but rather unsustainable way of DM disease management involves regular preventive and extensive application of fungicides. However, extensive fungicide use may have adverse environmental effects, leading to social disapproval, raising public concern about environmental issues, and high production costs (Wightwick et al. 2010; Marinho et al. 2020). In addition, the range of active compounds available for *P. viticola* regulation is steadily declining, as many products are losing their registration and only a limited number of new active ingredients are being approved. Furthermore, the efficacy of certain fungicides is diminishing due to the emergence of resistant *P. viticola* populations, with some products failing to provide adequate protection against newly arising strains (Massi et al. 2021). Continuous research has been conducted to develop less hazardous and more commercially viable fungicides. Nevertheless, one potential strategy involves the identification of novel resistances from non-adapted wild *Vitis* species for the effective and economic control of downy mildew as they allow to significantly reduce the amount of plant protection sprays (Schwander et al. 2012; Venuti et al. 2013; Sargolzaei et al. 2020; Eisenmann et al. 2023; Trapp et al. 2025; Petucco et al. 2026). The combination of loci with different genetic resistance mechanisms in a variety is crucial for the compelling and sustainable control of epidemics (Peressotti et al. 2010; Wingerter et al. 2021). Moreover, one of the new challenges and major consequences of climate change on viticulture is the intensified outspread of pathogens (Droulia and Charalampopoulos 2021; Töpfer and Trapp 2022).

Since the second half of the nineteenth century, wild *Vitis* species are used in the breeding of disease-resistant grapevine varieties due to their disease-resistant traits resulting from coevolution with the pathogens (Töpfer et al. 2011). Therefore, the ultimate goal of disease-resistance breeding is to generate new varieties with desirable combinations of resistance genes. Thus, over the years, grapevines have been bred effectively for disease resistance, allowing growers to reduce the use of fungicides worldwide while maintaining the desired qualities of *V. vinifera* cultivars (Eibach and Töpfer 2015). To date, 37 downy mildew resistance loci have been reported on various chromosomes, originating from diverse genetic backgrounds including American and Asian *Vitis* species as well as *V. vinifera* (Koledenkova et al. 2022). Well-known examples include *Rpv1* and *Rpv2* from *Muscadinia rotundifolia*, *Rpv3* from *Vitis rupestris*, several loci such as *Rpv5*, *Rpv6*, and *Rpv9* from *Vitis riparia*, and multiple loci (e.g., *Rpv8, Rpv10,* and *Rpv12*) from *Vitis amurensis* (Vezzulli et al. 2022; www.vivc.de/loci). These loci exhibit varying levels and mechanisms of resistance, reflecting the broad genetic diversity available for resistance breeding. Genetic markers associated with downy mildew resistance loci have been successfully utilized in marker-assisted selection (MAS), thus reducing the length of the breeding process by up to 10 years (Eibach and Töpfer 2015; Trapp et al. 2025). Additionally, some of these loci have been utilized in the development of new resistant cultivars and stacking resistance (Eibach et al. 2007; Merdinoglu et al. 2018; Zini et al. 2019; Sánchez-Mora et al. 2022; Reisch et al. 2023; Bitzsadze et al. 2024).

Apart from genetic resistance, the physical resistance displayed by leaf hair (trichome) density to prevent fungal infection due to its hydrophobic characteristics has been thoroughly investigated (Kortekamp and Zyprian 1999; Kono et al. 2018; Yin et al. 2021). In general, leaf hairs are widely distributed on the surface of different organs of the grape genus *Vitis* and is also utilized in ampelographic studies (Gago et al. 2016). Several studies have shown that leaf hair structures are effective in providing protection from herbivores, insects, and pathogens; regulating temperature; and preventing excessive water loss (Wagner et al. 2004; Ma et al. 2016). The association of leaf hair density with downy mildew disease resistance is of importance to breeders because it could serve as the first line of defense (Kortekamp et al. 1999). In the genus *Vitis*, few QTLs have been identified for leaf hair density (Barba et al. 2019; Kono et al. 2018; Yin et al 2021). Kono et al. (2018) were able to correlate the presence of leaf hair density with downy mildew resistance.

We observed effective field resistance to DM in the COxGT2 genotype derived from *Vitis coignetiae* Pulliat. Thus, the primary objective of this research was to phenotype *P. viticola* resistance in the ‘Morio Muskat’ × COxGT2 (*V. coignetiae* × ‘Gewürztraminer’) F1 population (*N* = 496) for three years under laboratory conditions. Furthermore, we aim to construct a rhAmpSeq marker-based genetic map for this population, and to perform a quantitative trait loci analysis (QTL) to identify the locus associated with DM resistance. Another aim was to develop molecular markers tightly linked to this locus to enable efficient selection through marker-assisted breeding in grapevine. Additionally, the biparental population showed segregation for the morphological trait leaf hair density. The resistant parent COxGT2 showed a high density of trichomes in contrast to the susceptible and almost hairless parent ‘Morio Muskat’. Therefore, another goal of this study was to phenotype this trait, perform QTL analysis and investigate the role of leaf hair density in resistance to DM.

## Material and methods

### Plant material

Investigations were carried out on a biparental segregating F1 population Gf.2018-063 (*N* = 496), which was derived from the cross of ‘Morio Muskat’ (VIVC 7996) × COxGT2 (VIVC 27137) (The numbers in parentheses after the variety names refer to the unique variety number in the VIVC database, www.vivc.de). The cross was carried out in 2018, the seeds germinated in the following year and the seedlings were cultivated in pots. ‘Morio Muskat’ is a *V. vinifera* cultivar with muscat flavor crossed at JKI Geilweilerhof in 1928. The breeding line COxGT2 (*V. coignetiae* × ‘Gewürztraminer’) was obtained from the nursery of grapevine breeder Edwin Schrank in Dackenheim, Germany. Parental genotypes and other cultivars, including ‘Kunbarat’ (VIVC 6557), ‘Müller Thurgau’ (VIVC 8141), ‘Solaris’ (VIVC 20340), and accessions of the wild species *V. amurensis* Ruprecht (VIVC 25953; *Rpv8* donor) and *V. coignetiae* Pulliat (accession SWR1989, VIVC 27870), were utilized in histochemical analysis and sporulation experiments. The resistance loci of the reference genotypes were based on published data and marker analysis performed in this study: the *V. amurensis* accession is reported to carry *Rpv8* (Blasi et al. 2011), ‘Kunbarat’ carries *Rpv12* (Venuti et al. 2013), and the resistant parent COxGT2 carries the newly identified locus *Rpv32* (this study). Marker analysis confirmed the presence of these resistance loci and their reciprocal absence in these genotypes. No additional loci like, e.g., *Rpv3* or *Rpv10* were found (Müllner 2021). All individual plants were potted and greenhouse-grown at the JKI Institute for Grapevine Breeding at the Geilweilerhof, Siebeldingen (49°13′05.0′′ N 8°02′45.0′′ E). Plant protection measures were taken to prevent powdery mildew infections, and no further plant protection was applied a week before inoculation experiments.

### Phenotyping for downy mildew resistance and leaf hair density

#### Inoculation experiment

To determine the resistance of leaves to DM in the F1 population Gf.2018-063, leaf disc assays were performed from 2020 to 2022. To standardize the physiological stage, two leaves from each individual were collected from the third and fourth apical nodes. Four leaf discs (one cm in diameter) were punched using a cork borer and placed upside down on a 1% agar water template (Insula AgarAgar Pulver E406; Gustav Essig GmbH and Co. KG, Mannheim, Germany) in 245 mm Square BioAssay Dishes (Corning®, New York, USA). Three of the four leaf discs were artificially infected by placing 30 μL of sporangia suspension droplets (approximately 22,000 sporangia/mL) on the leaf disc, while the fourth leaf disc was used as a control and treated with water. The parental lines as well as the susceptible *V. vinifera* cultivar ‘Müller Thurgau’ and resistant cultivar ‘Solaris’ were used as controls. After overnight incubation in the dark, the spore suspension droplets were removed the next day using a conventional water jet vacuum pump (aspirator) connected to a pipette tip to generate suction. The leaf discs were then incubated under full-spectrum light (16 h) at 20–23 °C and 72% relative humidity.

For inoculum preparation, spores of *P. viticola* were collected from grapevine varieties ‘Riesling’ (VIVC 10077) and ‘Müller Thurgau’ that were naturally infected in the vineyards of the JKI Institute for Grapevine Breeding. The inoculum therefore represented a mixture of pathogen genotypes and was not single-spored. The initial inoculum was maintained and propagated across experimental years on leaves of ‘Riesling’ and ‘Müller Thurgau’ under controlled laboratory conditions (23 °C, 72% relative humidity). When necessary, additional inoculum was collected from naturally infected field material to ensure sufficient spore availability. The harvested dry spore material was stored at −20 °C and thawed at room temperature immediately before use.

#### Automated image acquisition and manual phenotyping

**Fig. 1:**
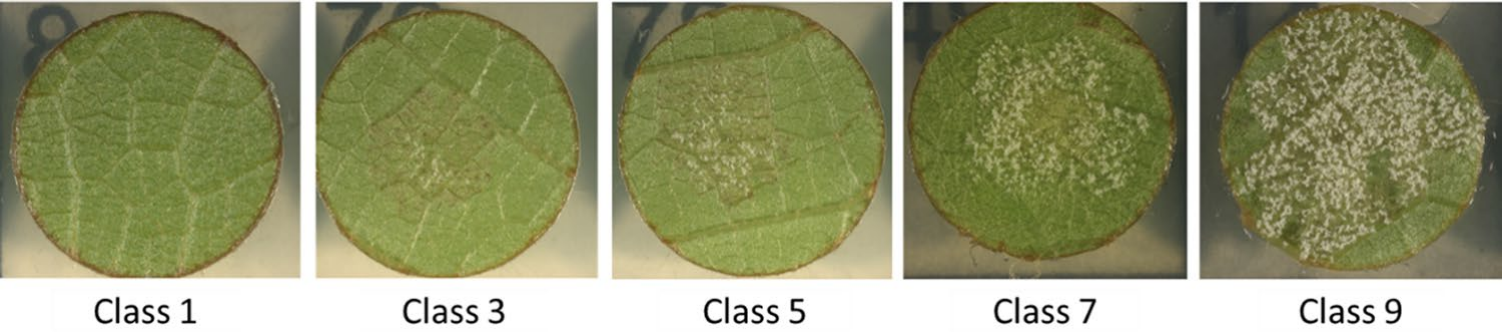
A reversed five-class OIV 452-1 descriptor for downy mildew evaluation

Five days after inoculation, images of the leaf discs were taken using a Zeiss Axio Zoom v16 microscope (Jena, Germany) equipped with a motorized stage. The images were captured with a 10.5-fold magnification and a 0.5 × magnification lens (PlanApo Z 0.5 × /0.125; Free Working Distance 114 mm). A carrier template generated with the ZenBlue (version 3.4; Zeiss, Jena, Germany) software was used to automatically acquire images of 96 leaf discs on a single plate. The images of the leaf discs (2752 × 2208 pixels) were saved in JPEG format (Zendler et al. 2021) and later manually evaluated using the reversed five-class OIV 452-1 descriptor (OIV 2009). DM disease severity was evaluated based on the intensity of sporangia formed on the leaf discs. The severity was scored on a scale of 1–9, where score 1 indicated no sporangiophores (highly resistant), score 3 indicated one to five sporangiophores (resistant), score 5 indicated six to twenty sporangiophores (moderate), score 7 indicated more than twenty sporangiophores (susceptible), and score 9 indicated a dense cloud of sporangiophores (highly susceptible) (Fig. 1). In total, seven experiments were conducted over three years.

**Fig. 2:**
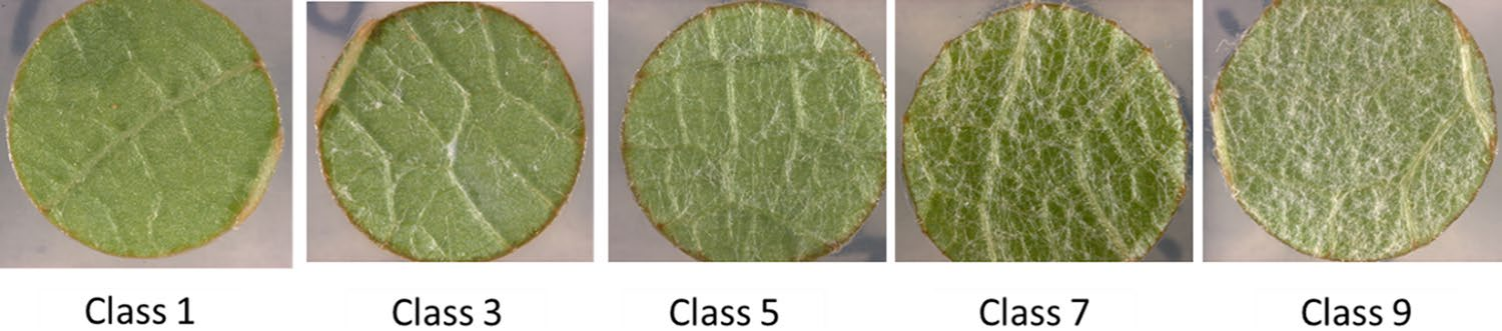
Five-class OIV 084 descriptor for leaf hair density

Leaf hair density was assessed on leaf discs derived from the third and fourth fully expanded leaves from the shoot apex, ensuring a comparable developmental stage. Leaf size was not quantified as a percentage of full expansion, but only fully expanded leaves were selected for analysis. Similar to the procedure described above, the images of untreated leaf discs were visually assessed for their leaf hair density utilizing the OIV descriptor 084 (OIV 2009). The classes included in this assessment were 1: none/very low, 3: low, 5: medium, 7: high, and 9: very high density of leaf hair (Fig. 2). For each genotype, four leaf discs were evaluated. A single phenotypic evaluation was conducted each year from 2020 to 2022, resulting in three datasets. For correlation analysis, mean phenotypic values per genotype across experiments were used.

#### Aniline blue staining

The study of intracellular hyphal growth was conducted at 1, 3, and 5 days post-inoculation (dpi) in the leaves of the parental genotypes. For each genotype at each time point, three leaf discs were analyzed. The discs were incubated in 1 M KOH for 3–6 h at 65 °C and subsequently rinsed in deionized water (dH_2_O). Treated discs were placed upside down on a glass slide and stained with aniline blue (0.05%) in K_2_HPO_4_ (0.067 M) for 10 min. After rinsing with dH_2_O, excess solution was removed by gently blotting with a paper towel. The stained hyphae were observed under a fluorescence microscope (Zeiss Axio Zoom V16, excitation *λ* = 450–490 nm), and images were captured in JPEG format. Mycelium growth was assessed qualitatively based on visual examination of the captured images; no quantitative measurements were performed.

#### Growth and sporulation of *P. viticola*

To determine the level of plant resistance to *P. viticola*, pathogen growth and development within plant tissue were observed and quantified five days post-inoculation. In addition to the parental lines ‘Morio Muskat’ and COxGT2 (*Rpv32*), a susceptible genotype ‘Müller Thurgau’ and three *P. viticola* resistance carrying genotypes, namely *V. amurensis* (*Rpv8*), ‘Kunbarat’ *(Rpv12*), and *V. coignetiae* (*Rpv32*), were included in the experiment. The two genotypes originating from Asia carrying *Rpv8* and *Rpv12,* respectively, were of particular interest. After five days of inoculation, each leaf disc was washed in an Eppendorf tube. If necessary, any remaining pathogen on the leaf discs was removed using a needle, and the washing process was repeated. The tubes were then vortexed, and the sporangia were observed and counted using a hemocytometer. The average of six leaf discs was used in the statistical analysis. Multiple pairwise comparison tests were conducted to compare the differences between the various genotypes.

### Genotyping

#### DNA extraction and rhAmpSeq marker analysis

Genomic DNA was extracted from young and healthy grapevine leaf material. Approximately 1–2 cm^2^ of leaves were collected from the third leaf of the shoot tip. The harvested leaf material was placed in 96 deep well plates containing steel beads, frozen in liquid nitrogen, and ground using a TissueLyser mill (Qiagen GmbH, Hilden, Germany). PeqGOLD Plant DNA Mini Kit (VWR, Darmstadt, Germany) was used to isolate genomic DNA. The extracted DNA concentration and quality were checked by spectrophotometric measurement (CLARIOstar, BMG Labtech, Ortenberg, Germany) at 260 nm (A260). To prepare for rhAmpSeq (RNase H2-dependent amplicon sequencing markers) marker analysis, the average concentration of each DNA sample was adjusted to 1 ng/μL by dilution before it was shipped to Cornell University (Geneva, New York, USA) on dry ice in 2020 for analysis. Approximately 2000 amplicon-based haplotype markers were tested on the population Gf.2018-063 and a raw output file was provided called ‘hap genotype’. This file was filtered using the Python script slice.py to remove variants with a minor allele frequency below 0.05, followed by conversion to Variant Call Format using the Perl script to_lep_map.pl, both provided by Cornell University as part of the rhAmpSeq analysis pipeline (Zou et al. 2020; Karn et al. 2021).

#### Genetic mapping and QTL analysis

As a first step, quality control (QC) analyses (Mendelian error, selfing, cross contamination) were performed using TASSEL 5.0. The mapping and QTL analyses did not include QC test failed samples. To determine QTLs, a genetic map for the population Gf.2018-063 was generated using Lep-MAP3 v.0.2 software (Rastas 2017, https://sourceforge.net/p/lep-map3/wiki/LM3%20Home/). Lep-MAP3 constructs parental phase-informed linkage maps prior to generating the integrated map, thereby accounting for parent-specific recombination patterns. All the following Lep-MAP3 modules were implemented to generate a rhAmpSeq-based linkage map: (1) ParentCall2: used to call parental genotypes; (2) Filtering2: for filtering (dataTolerance = 1.00E-6) the distored/monomorphic markers based on a chi-square (*χ*^2^) test; (3) SeparateChromosomes2 module: used to split the markers into respective linkage groups (LG = 19); (4) OrderMarkers2module: used to order the LG-specific markers (iterations/group = 20). Based on the markers’ genetic positions (cM) and physical positions in the reference genome (PN40024 12X.v2) (Zou et al. 2020), marker collinearity was estimated to check for genome organization and structural variation. Using the ‘map2genotypes.awk’ script, phased output data were converted into phased genotype data for QTL analysis. A four-way cross QTL mapping was performed in R/qtl using the Kosambi mapping function (R software, version 3.4.5; R Core Team 2020). Genotype probabilities were calculated with a step size of zero centimorgans, no extension beyond the ends of the LGs, a genotyping error probability of 1.0 × 10^−4^, and a fixed step width. Composite interval mapping was conducted using the Kosambi mapping function. Genome-wide significance thresholds at *α* = 0.05 were determined by 1,000 permutations, and the resulting logarithms of the odds (LOD) threshold was applied to identify significant QTL. Confidence intervals for detected QTL were defined using the 1.8-LOD drop method with a probability of coverage of 0.95. Genotype simulations for subsequent analysis were performed with 32 draws, applying the same mapping function and error parameters as in the genotype probability calculation.

#### SSR marker-based comparison of *Rpv32* with *Rpv8* and *Rpv12*

Microsatellite analysis was conducted utilizing published simple sequence repeat (SSR) primer pairs as reported by Blasi et al. (2011) for *Rpv8* and by Venuti et al. (2013) for *Rpv12*. These primer sets were evaluated on three genotypes: *V. amurensis* carrying *Rpv8*, the cultivar ‘Kunbarat’ carrying *Rpv12*, and the resistant parent COxGT2 carrying *Rpv32*. Multiplex PCRs were executed using fluorescently labeled primers (TAMRA, ROX, HEX, 6-FAM) attached to the 5′-end of the forward primers (Metabion, Planegg, Germany). Reactions were prepared in 5 µL volumes with the Qiagen Multiplex PCR Kit (Qiagen, Hilden, Germany), containing approximately 1 ng of genomic DNA and 3 pmol of each forward and reverse primer. The amplification conditions were as follows: initial denaturation at 95 °C for 180 s; 30–35 cycles of 95 °C for 15 s, 60 °C for 30 s, and 72 °C for 30 s; followed by a final elongation step at 72 °C for 560 s. PCR products were diluted 1:2 with dH_2_O and analyzed via capillary electrophoresis using an ABI PRISM 3130xl Genetic Analyzer (Applied Biosystems, Darmstadt, Germany) with GeneScan 500 LIZ as the internal size standard. Allele sizes were determined using GeneMapper Version 5, and allele scoring was performed as described by Müllner (2021). Since the names of the rhAmpSeq markers refer to the physical position based on the reference genome PN40024 12X.v2, the physical positions of the SSR markers were also referenced to PN40024 12X.v2 (Canaguier et al. 2017) to ensure comparability.

## Results

### Downy mildew phenotypic data

**Fig. 3:**
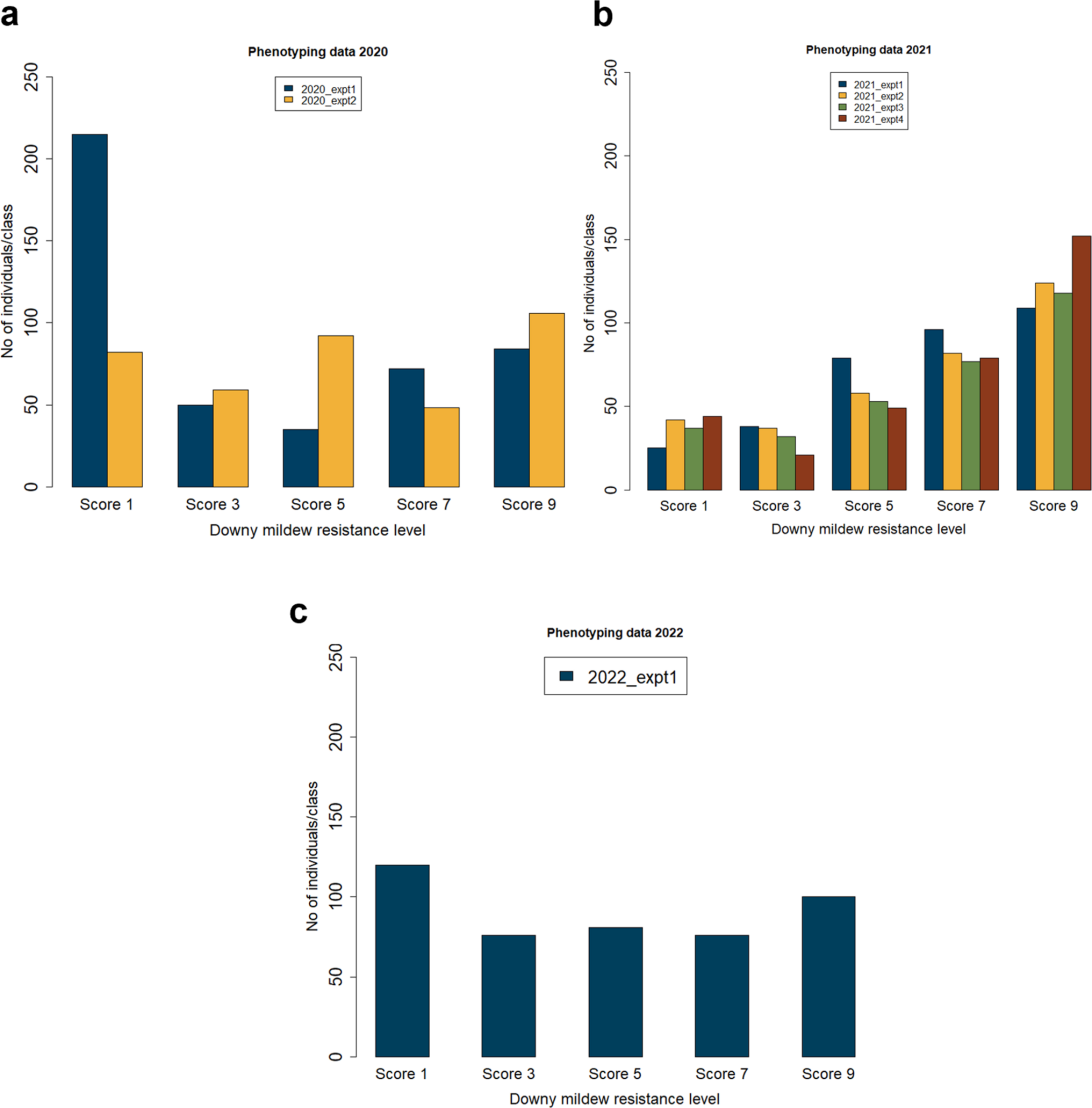
Phenotypic distribution of *P. viticola* resistance (3a, b, c) in the Gf.2018-063 population over three years. Resistance was scored on leaf discs using the reversed OIV 452-1 scale (1 = highly resistant, 9 = highly susceptible). Panels show results for 2020 (3**a**), 2021 (3**b**) and 2022 (3**c**). X-axis: score class; y-axis: number of individuals. Colors indicate individual experiments (3a, b, c) (color figure online)

The biparental population Gf.2018-063 was subjected to leaf disc assays in a laboratory setting to evaluate DM resistance and leaf hair density. For the purpose of assessing DM resistance, leaf discs were manually evaluated using the reversed OIV 452-1 descriptor (Fig. 1). Seven phenotypic evaluations were performed in three years. The distribution of phenotypic data and trait segregation for the years 2020 (comprising two experiments), 2021 (consisting of four experiments), and 2022 (covering a single experiment) are depicted in bar plots (Fig. 3a, b, c). Although the phenotypic distributions varied between years and individual experiments, this variation is to be expected in controlled infection assays and may reflect differences in experimental conditions. Notably, some variability was observed between replicates in 2020. However, the overall segregation pattern remained consistent over the years. In every experiment, the susceptible parent ‘Morio Muskat’ displayed severe downy mildew symptoms on the leaf discs (class 9), while the resistant parent COxGT2 showed low disease severity (classes 1–3). Phenotypic distributions and corresponding DM resistance QTL curves are color-coded consistently to allow straightforward comparison.

### Genetic map

The Gf.2018-063 population (N = 496) was genotyped with 2000 rhAmpSeq markers. After quality control filtering, 41 individuals and low-quality markers were removed, resulting in 455 individuals genotyped with 639 informative markers (32% of the initial set). A total of 19 LGs were constructed, spanning 1147.3 cM with an average of 45 loci per LG. LG12 contained the fewest markers (12), whereas LG07 contained the most (49). The genetic length of individual LGs ranged from 46.0 cM (LG15) to 70.4 cM (LG01). Collinearity analysis between genetic and physical positions, based on the PN40024 reference genome (12X.v2), showed high overall correlation (Pearson’s r > 0.90) (Supplementary Fig. S1). Nevertheless, discrepancies were detected on several chromosomes. In particular, markers on the upper arm of chromosome 14 did not follow the expected physical order, and the interval between 0.088 and 19.1 Mb was represented by a short genetic distance, indicating suppressed recombination and/or structural divergence between the *V. coignetiae* derived haplotype and PN40024. Similar inconsistencies were also observed on chromosomes 3, 10, 12, and 17. As indicated by Zou et al. (2020), structural variation and genome organization inconsistencies are more prevalent in wild *Vitis* genotypes than in *V. vinifera*, which may explain the observed physical gaps. The complete rhAmpSeq-based genetic map of the Gf.2018-063 population is shown in Supplementary Fig. S2.

### QTL analysis: Downy mildew resistance

**Fig. 4:**
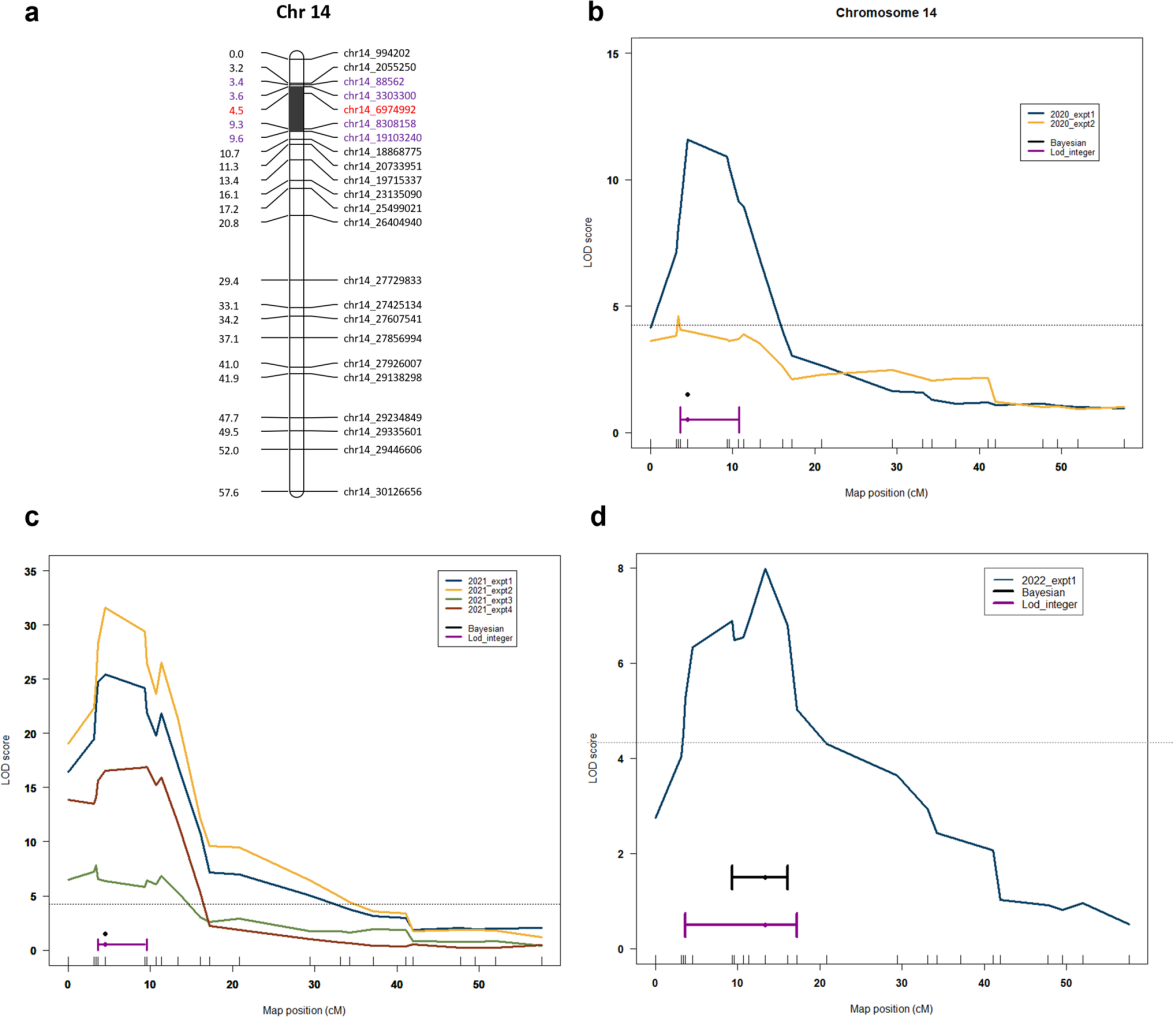
QTL analysis for DM resistance. **a** The genetic position of the peak marker (bold red) and their LOD interval (marked purple) on the upper part of chromosome 14 (Chr 14). LOD score for genetic markers distributed across the Chr 14 for the individual experiment year **b** 2020, **c** 2021 and **d** 2022. The dotted line (black) indicates the 95% confidence threshold. ‘2020_expt1’ indicates the year and the number of the independent experiment conducted in ascending order. Bayesian (black colored interval) and LOD integer (purple colored interval)-based interval are shown separately (color figure online)

Over the course of multiple years, a consistent and strong QTL has been identified in all phenotypic data sets (Fig. 4, Table 1). The QTL was found to account for up to 28.16% of the phenotypic trait variance and was designated as ‘*Rpv32*’. In 2020, the QTL was detected in two experiments, yielding LOD values of 11.6 and 4.6, which accounted for 11.5 and 4.7% of the total phenotypic variance, respectively, within a 3.2 and 4.5 cM region (Fig. 4b). In 2021, all experiments have consistently yielded the same QTL, with improved LOD values ranging from 7.82 to 31.6 and variance explained ranging from 7.8 to 28.16% (Fig. 4c). The DM trait was significantly correlated with the LODmax marker chr14_6974992 at 4.5 cM, and the locus was consistently delimited by the flanking markers: on the left, chr14_88562 (3.4 cM) and chr14_3303300 (3.6 cM); on the right, chr14_8308158 (9.3 cM) and chr14_19103240 (9.6 cM) (Fig. 4a). In 2022, the QTL was again detected (Fig. 4d), further confirming the presence of natural genetic resistance to DM in COxGT2 (see Supplementary Fig. S3). Across all three years, the QTL was consistently located within this interval, defined by the left flanking markers chr14_88562/chr14_3303300 and the right flanking markers chr14_8308158/chr14_19103240 (Table 1). Despite the observed variability in phenotypic data between years and experiments, the QTL on chromosome 14 was consistently detected across all datasets, supporting the robustness of the identified resistance locus. In addition to the rhAmpSeq markers, three SSR markers located within the *Rpv32* interval have been validated in COxGT2 and can be reliably employed to track the resistance allele in breeding populations (Supplementary Table S1). These SSR markers offer immediate tools for MAS.

**Table 1:** Summary of the QTL analysis for resistance to DM in population Gf.2018-063 for the years 2020, 2021 and 2022

Trait: Downy mildew resistance (Chr 14)						
Year	LODmax marker [bp]	Peak marker position [cM]	LOD value	PVE [%]	Left flanking marker	Right flanking marker
2020	**chr14_6974992**	4.5	11.6	11.56	chr14_3303300	chr14_18868775
	chr14_88562	3.4	4.61	4.77	chr14_994202	chr14_23135090
2021	**chr14_6974992**	4.5	25.42	23.32	chr14_88562	chr14_19103240
	**chr14_6974992**	4.5	31.6	28.16	chr14_3303300	chr14_8308158
	chr14_88562	3.4	7.82	7.83	chr14_994202	chr14_19715337
	chr14_19103240	9.57	16.9	16.37	chr14_88562	chr14_19715337
2022	chr14_19715337	13.4	7.98	8.13	chr14_3303300	chr14_25499021

The shared LODmax marker is highlighted in bold. The LOD value indicates the LOD score at the peak position (LODmax marker); PVE represents the phenotypic variance explained (%). Physical positions of the rhAmpSeq markers are given in base pairs [bp] according to the PN40024 reference genome (12X.v2). The genome-wide significance thresholds (4.2) were determined with 1000 permutations (*α* = 0.05). Left and right flanking markers for each LODmax marker are indicated

### *Rpv32* represents a novel resistance locus different from *Rpv8* and *Rpv12*

Previously, two other DM resistances have been mapped in the genomic region of *Rpv32* on chromosome 14: *Rpv8* and *Rpv12* (Blasi et al. 2011; Venuti et al 2013). Both resistances originate from the East Asian species *V. amurensis*, which, like *V. coignetiae*, is also native to East Asia. To rule out a possible genetic relationship of *Rpv32* to *Rpv8* and *Rpv12*, genotypes carrying *Rpv8* (*V. amurensis*), *Rpv12* (‘Kunbarat’), and *Rpv32* (COxGT2) were genotyped with eight SSR markers located in the genomic region of these loci. The comparison of the SSR marker results confirmed that the allele sizes observed in the *Rpv32* carrying genotype COxGT2 were distinct from those associated with *Rpv8* and *Rpv12*. These findings demonstrate that the COxGT2 and *V. amurensis* accessions do not share a common ancestry at these loci, providing strong evidence that *Rpv32* represents a novel resistance locus independent of *Rpv8* and *Rpv12* (Table 2).

**Table 2:** Comparative SSR marker analysis of *Rpv32* with published markers linked to *Rpv8* (*V. amurensis*) and *Rpv12* (‘Kunbarat’)

SSR marker	Physical position 12X.v2[bp]	*V. amurensis* (*Rpv8*)	Kunbarat (*Rpv12*)	*V. coignetiae* (*Rpv32*)
Chr14V015	6,641,772	212	212	207
Sc81_7.4	8,426,890	275	275	322
Sc81_8.2	8,741,491	null	265	null
UDV-350	8,963,923	308	310	320
UDV-343	9,012,000	160	160	172
UDV-345	9,058,144	220	220	216
UDV-340	9,145,662	178	178	176
UDV-360	9,910,488	208	208	184

The physical positions of the SSR markers are given according to the PN40024 reference genome (12X.v2). Allele sizes (bp) are shown for *V. amurensis* (*Rpv8*), ‘Kunbarat’ (*Rpv12*), and COxGT2 (*Rpv32*). Distinct allele sizes observed in COxGT2 demonstrate that *Rpv32* is genetically independent of *Rpv8* and *Rpv12*. null: null allele

### Comparison of *P. viticola* sporulation among genotypes

**Fig. 5:**
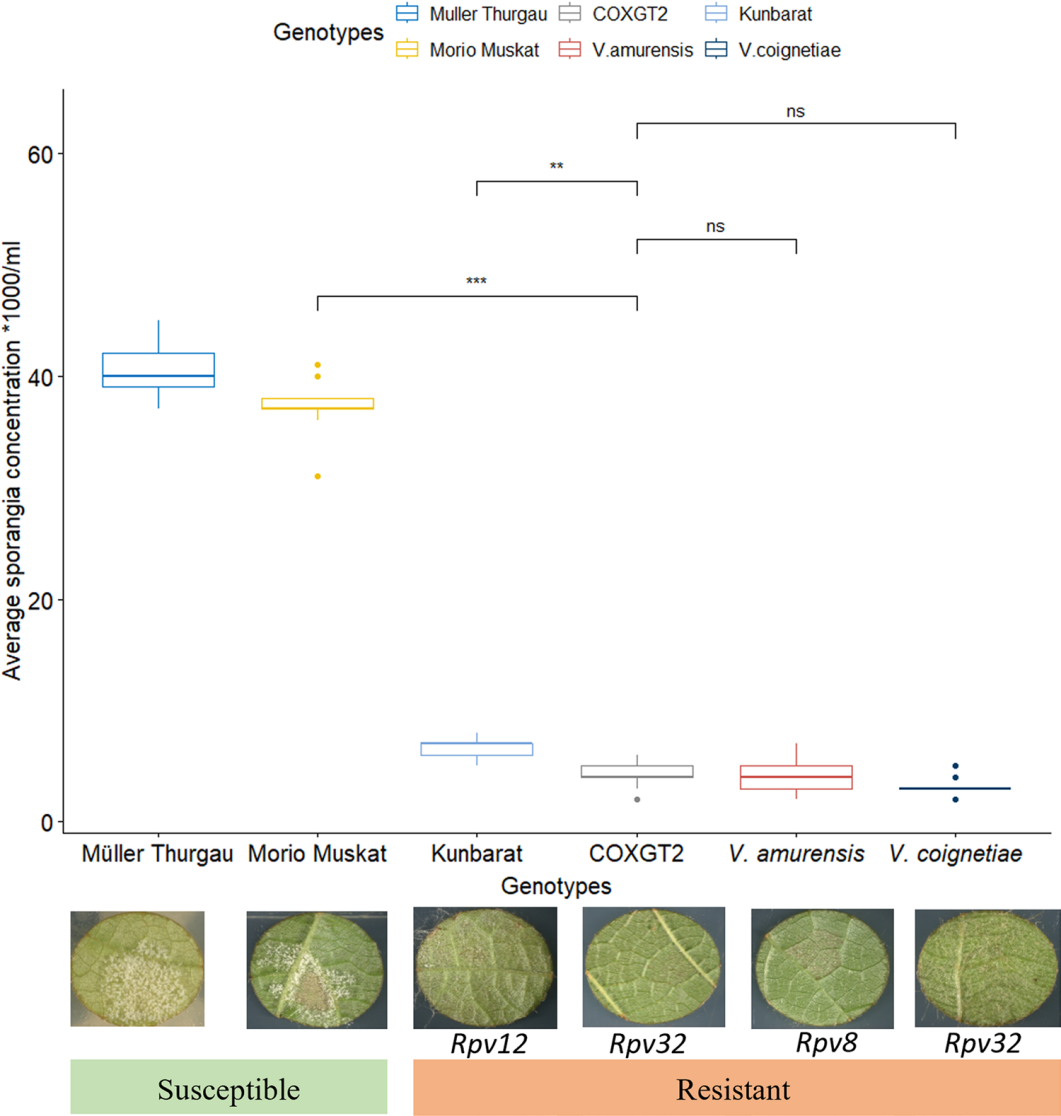
*Plasmopara viticola* sporulation in susceptible (‘Müller Thurgau’ and ‘Morio Muskat’) and resistant (COxGT2, ‘Kunbarat’, *V. amurensis,* and *V. coignetiae*) genotypes on 5dpi. Whiskers box plot indicating multiple pairwise comparisons using Kruskal–Wallis and Conover–Iman test (p < 0.05). X-axis: Genotypes and their respective resistance loci are indicated below the image. Y-axis: average sporangia concentration per mL. ns: non-significant. *Rpv:* Resistance to *P. viticola*. The number of observations per genotype is *N* = 6 leaf discs

To quantify the proliferation of *P. viticola* on parental and different resistant genotypes, the sporangia formed on infected leaf discs were counted at 5 dpi. Two susceptible *V. vinifera* cultivars, ‘Müller Thurgau’ and ‘Morio Muskat’ (parent), as well as resistant genotypes carrying *Rpv8* (*V. amurensis*), *Rpv12* (‘Kunbarat’), and *Rpv32* (COxGT2 and *V. coignetiae*) were included. Multiple pairwise comparison (Kruskal–Wallis and Conover-Iman test) between the genotypes demonstrated significant and reliable differences between the susceptible (~ 40,000 sporangia/mL) and all the resistant (~ 2000–5700 sporangia/mL) genotypes (Fig. 5). In general, the number of sporangia quantified in the COxGT2 (*Rpv32*) genotype showed significantly lower sporulation than the susceptible parent. The level of sporulation in COxGT2 was significantly lower in contrast to ‘Kunbarat’ (*Rpv12*). Furthermore, no substantial differences in sporangia counts were found between COxGT2 and *V. amurensis* (*Rpv8*). Interestingly, there were no significant differences in sporulation between the F1 (COxGT2) and the parent of the F1, *V. coignetiae*, illustrating no obvious loss of resistance after crossing.

### Histochemical analysis

**Fig. 6:**
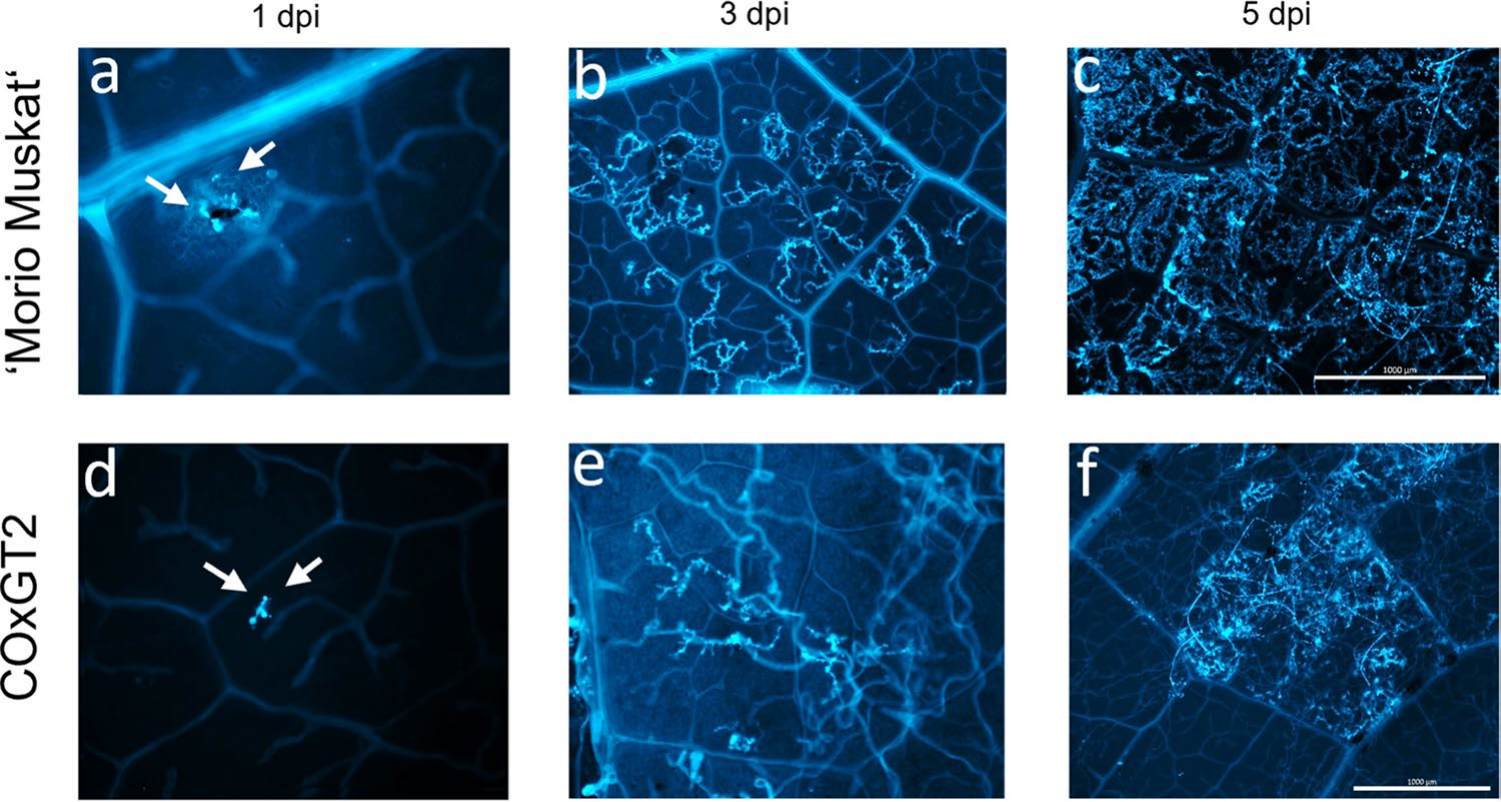
Histochemical analysis of *P. viticola* infected leaf discs in parental genotypes after aniline blue staining (Susceptible: ‘Morio Muskat’; Resistant: COxGT2) at different time points at 1, 3, and 5 dpi. Leaf discs were stained with aniline blue and visualized under fluorescence microscopy (excitation *λ* = 450–490 nm). **a**, **d** Early infection structures (arrows) observed at 1 dpi. **b**, **e** Hyphal expansion at 3 dpi. **c**, **f** Extensive mycelial growth in ‘Morio Muskat’ compared with restricted development in COxGT2 at 5 dpi. Insets in **a** and **d** show magnified views of infection structures. Scale bars: 100 µm (a, d), 500 µm (b, e), and 1000 µm (c, f) (color figure online)

The present study utilized aniline blue-based histochemical analysis to investigate the intracellular development of the mycelium network in *P. viticola*-inoculated leaf discs of susceptible ‘Morio Muskat’ and resistant COxGT2. At different time points (1, 3, and 5 days), three independent experiments were conducted, and representative images are depicted in Fig. 6. On the first day, the hyphae and germ tubes developed similarly in both susceptible and resistant genotypes. However, on day 3, substantial differences were observed between the two genotypes. The susceptible parent displayed moderate hyphal growth with pronounced extension of primary hyphae, whereas the resistant parent showed restriction of primary hyphae and overall mycelium growth. On day 5, the susceptible genotype exhibited extreme proliferation, covering the entire tissue, while the resistant genotype experienced hindrance to the development process. Additionally, on day 5, sporangiophores were fully visible on the abaxial surface of both parental genotypes. Although the resistant parent showed limited pathogen growth at the early stage (1 dpi), it was able to contain the infection and prevent further spread by 3 dpi. Collectively, these findings indicate that pathogen growth and development were initiated within the first 1–3 days but were subsequently restricted in the resistant parent.

### Leaf hair density: Phenotypic data and QTL analysis

**Fig. 7:**
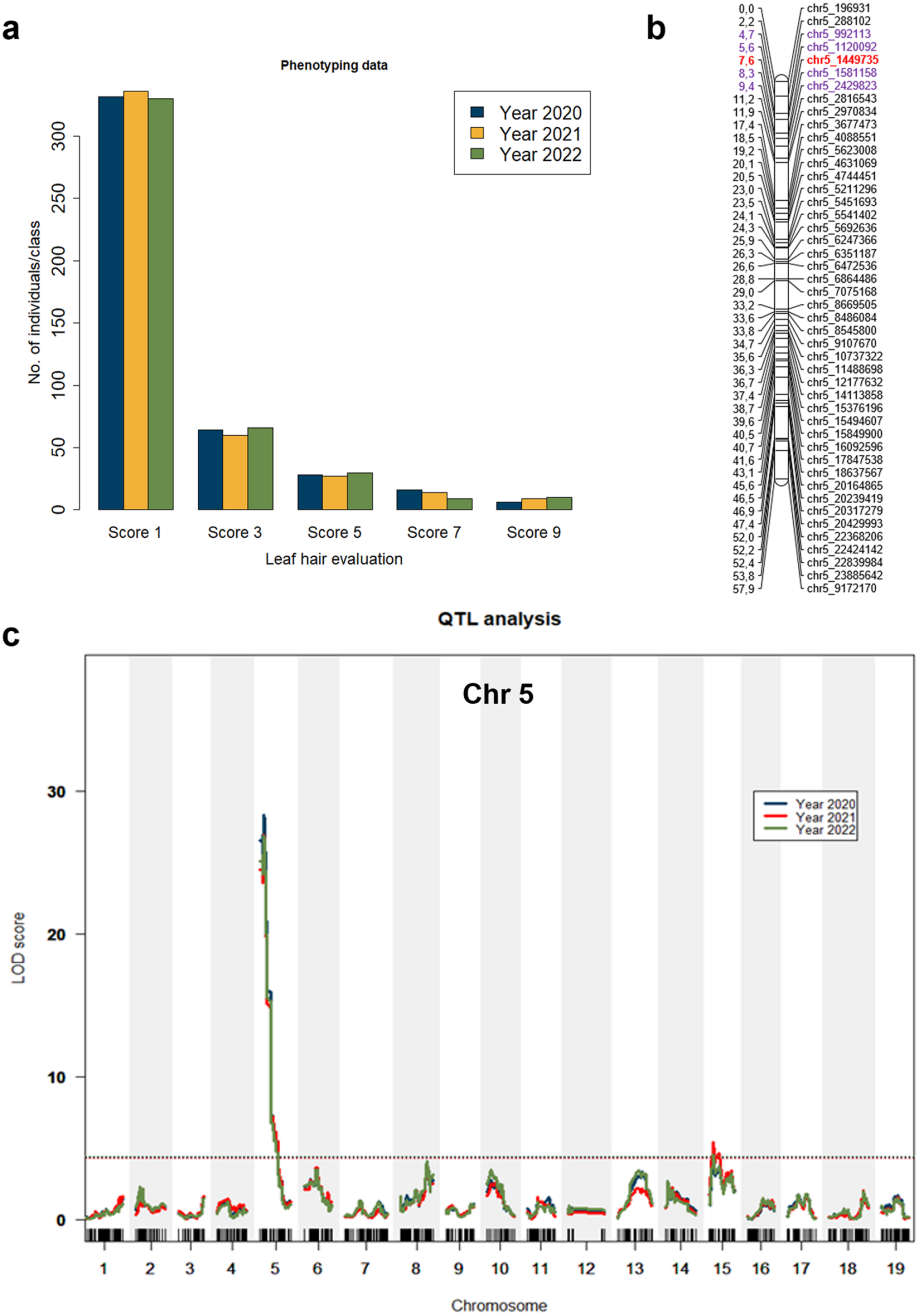
QTL analysis for leaf hair density. **a** Phenotypic distribution of leaf hair density in the Gf.2018-063 population across 2020, 2021, and 2022, scored according to OIV 084 (1 = none/very low, 9 = very high). Bars show the number of individuals per score class. **b** Genetic map of chromosome 5 (Chr 5) indicating the peak marker (bold red) and the flanking markers (purple) delimiting the 1.8-LOD confidence interval; genetic positions (cM) are shown on the left and physical marker positions (bp, PN40024v2 reference) on the right side. **c** QTL profiles for leaf hair density across the 19 chromosomes for 2020 (blue), 2021 (red), and 2022 (green). The x-axis shows the map position, the y-axis the LOD score, and the black dotted line represents the 95% confidence threshold (color figure online)

To evaluate the leaf hair density in the segregating population Gf.2018-063, an individual experiment was conducted each year (2020, 2021, and 2022). The distribution of phenotypic data for the three years is shown in a bar plot (Fig. 7a). The parent ‘Morio Muskat’ exhibited no hairs on the leaf discs (class 1), while the parent COxGT2 displayed a high density of leaf hairs (class 9). QTL analysis identified a single major locus responsible for leaf hair density on the upper arm of chromosome 5, spanning from 4.3 to 8.7 cM (1.8-LOD confidence interval). It accounted for 24.23–25.36% of the phenotypic trait variance in the years 2020, 2021, and 2022). The QTL displayed consistent and stable expression across all phenotypic data sets from these three years, as demonstrated in Fig. 7 and Table 3. The allele responsible for the trait leaf hair density was contributed by the maternal hairless parent ‘Morio Muskat’ as shown in an effect plot (Supplementary Fig. S4).

**Table 3:** Summary of the QTL analysis for leaf hair density in population Gf.2018-063 for the years 2020, 2021 and 2022

Trait: Leaf hair (Chr 5)						
Year	LODmax marker [bp]	Peak marker position [cM]	LOD value	PVE [%]	Left flanking marker	Right flanking marker
2020	chr5_1449735	7.63	28.33	25.36	chr5_196931	chr5_2429823
2021	chr5_1449735	7.63	26.92	24.26	chr5_1120092	chr5_2429823
2022	chr5_1449735	7.63	26.88	24.23	chr5_196931	chr5_2429823

QTL detection was performed using multiple imputation and the Haley–Knott (HK) method implemented in the R/qtl package, with 95% Bayesian confidence intervals. The LOD value indicates the LOD score at the peak position (LODmax marker); PVE represents the phenotypic variance explained (%). Physical positions of the rhAmpSeq markers are given in base pairs [bp] according to the PN40024 reference genome (12X.v2). The genome-wide significance thresholds (4.2) were determined with 1000 permutations (*α* = 0.05). Left and right flanking markers for each LODmax marker are indicated

### Correlation between downy mildew resistance and leaf hair density

It is assumed that high leaf hair density can play a critical role in preventing infections caused by *P. viticola*. Therefore, the influence of leaf hair density on genetic resistance was investigated by correlating three years of manually evaluated leaf hair phenotypic data with downy mildew resistance phenotypic data for each year. The Pearson’s correlation coefficient (r) ranged from − 0.08 to − 0.12 for all three years. The association between downy mildew and leaf hair density was found to be relatively weak, rather than the fact that it was negatively correlated.

## Discussion

Downy mildew is one of the most destructive diseases affecting viticulture worldwide. From a breeding perspective, in order to ensure sustainable viticulture and minimize fungicide requirements, breeders initiated exploring and introducing naturally occurring resistances into the gene pool of *V. vinifera* (Töpfer and Trapp 2022). To date, 37 *Rpv* resistance loci have been identified in North American and Asian *Vitis* species. At least ten *Rpv* loci have been mapped in East Asian species, mainly in *V. amurensis* (Vezzulli et al. 2022). Nevertheless, the genetic diversity of Asian *Vitis* remains comparatively less explored than that of North American species, and only a limited number of these loci have been introgressed into the *V. vinifera* gene pool (Koledenkova et al. 2022; Vezzulli et al. 2022). *Rpv10* and *Rpv12*, which originate from Asian *V. amurensis*, are two of the most important loci currently utilized in grapevine breeding (Schneider et al 2019; Töpfer and Trapp 2022; Tomaz et al. 2025). Researchers have recently discovered several resistance loci against downy mildew in the varieties ‘Shuanghong’ and ‘Shuangyou,’ both descendants of the wild grape *V. amurensis*, named *Rpv22*, *Rpv23* and *Rpv24* (Fu et al. 2020), and *Rpv25* and *Rpv26* (Lin et al. 2019). Generally, Asian species such as *V. amurensis* did not co-evolve with the grapevine downy mildew pathogen *P. viticola*. Nevertheless, they exhibit resistance, which has been regarded as unexpected in the absence of a co-evolutionary history (Delmotte et al. 2014). *Vitis coignetiae* (crimson glory vine) is native to East Asia (mainly Japan, Korea, and parts of Russia), where it is cultivated both as an ornamental plant and, to a lesser extent, for winemaking. However, compared to *V. vinifera* or *V. labrusca* hybrids, its commercial importance is limited, and there is still little knowledge about the genetic basis of disease resistance in this species. In this research, a highly significant and novel QTL was identified that confers strong resistance to grapevine downy mildew from the unexploited East Asian species, *V. coignetiae*. The resistance locus was identified in a biparental F1 population derived from the *V. coignetiae* background (ancestor) in three different years. A QTL analysis approach was applied to 496 F1 individuals with 2000 rhAmpSeq markers. Genetic map construction and phenotypic data on resistance gained by leaf disc assays serve as a strong and proven basis for QTL analysis in grapevine (Welter et al. 2007; Bellin et al. 2009; Bhattarai et al. 2021). The recent approach of constructing dense genetic maps utilizing highly transferable and cost-efficient core *Vitis* genome rhAmpSeq markers has proven to be extremely successful (Zou et al. 2020, 2023; Karn et al. 2021; Alahakoon et al. 2022).

In accordance with prior genetic maps, this map presents comparable genome coverage across the 19 LGs (Sapkota et al. 2019, 2023; Reshef et al. 2022; Park et al. 2022), providing a dependable foundation for QTL analysis. However, only 643 (30%) markers yielded high quality data after quality control for the Gf.2018-063 population, which may be due to genotyping errors and low marker transferability resulting from the different genetic background of the resistant parent. In 2020, Zou et al. discussed the occurrence of parent or family specific anomalies. Additionally, they stated that mismatches in primers due to hemizygosity can contribute to failed PCR amplification. Similarly, in 2021, Karn et al. reflected on the role of genotyping errors and structural variations in decreasing marker numbers. Nonetheless, in this study, the average Pearson’s correlation achieved between the physical (PN40024 12X.v2) and genetic position is r > 0.90.

The QTL responsible for the phenotypic trait variance in question is located on chromosome 14 at the position of 4.5 cM (corresponding to 6,974,992 bp of PN40024 12x.v2). This QTL accounts for up to 28.16% of the variance in independent experiments conducted over three years (2020, 2021, and 2022). Although phenotypic distributions appeared relatively stable across experiments in 2021, variation in LOD values is expected and may result from differences in phenotypic variance, environmental conditions, and experimental noise, which influence the statistical power of QTL detection. Importantly, the consistent detection of the QTL across all experiments confirms the robustness of the identified locus. In the leaf disc assays, the susceptible parent ‘Morio Muskat’ exhibited high disease severity (reversed OIV 452-1, class 9), while the resistant parent showed very few sporangia (classes 1–3). The phenotypic data from the F1 individuals of population Gf.2018-063 supports the presence of a single major locus contributing to the resistance to *P. viticola*. This novel resistance locus, which originates from *V. coignetiae*, has been designated as ‘*Rpv32*’. The identification of 37 resistance loci (*Rpv*) to *P. viticola* has been a significant achievement in the field of viticulture. Of these, three loci have been located on chromosome 14, which seems to be a critical region for disease resistance in grapevines. Blasi et al. (2011) and Venuti et al. (2013) have mapped the Asian *V. amurensis* accession-derived resistances *Rpv8* and *Rpv12* on the upper arm of chromosome 14. *Rpv8* is located close to the ‘Chr14V015’ marker, while *Rpv12* is confined between the UDV014 and UDV370 markers. Although *V. amurensis* and *V. coignetiae* originate from the same continent, their different geographical origins raise some concerns about their characterization. To address this issue, two approaches were used. Firstly, Galet’s (1988) ampelographic characterization clearly distinguished between the shoots, young leaves, and mature leaves of *V. amurensis* and *V. coignetiae*. Secondly, the genetic analysis performed utilizing SSR markers published by Blasi et al. (2011) and Venuti et al. (2013) with genotypes carrying *Rpv8*, *Rpv12* and *Rpv32* showed significant and reliable differences in the SSR allele sizes between the resistance loci of *V. amurensis* and *V. coignetiae* (Table 2). In addition, the comparative SSR marker data of *Rpv8* and *Rpv12* are in well accordance with those of Müllner (2021). These results provide strong evidence that the *V. amurensis* accessions and COxGT2 (F1 of *V. coignetiae*) do not share any common ancestry and *Rpv32* is a novel resistance locus independent from *Rpv8* and *Rpv12*. The localization of *Rpv32* on chromosome 14 is further supported by SSR marker analysis, which independently confirms the position of the resistance locus.

Histochemical analysis was performed to observe the intracellular development of mycelium at different time points between the susceptible and resistant parental lines (Fig. 6). Between the parental lines, the aniline blue staining revealed no clear differences in growth at day 1 post-inoculation with *P. viticola*. The establishment of the infection by penetration of the pathogen into the stomata and the development of primary hyphae was very similar in the parental lines at 1 dpi. In general, these results confirm the observations reported in previous studies for other *Rpv* loci (Loon et al. 2006; Eisenman et al. 2019). The results at 3 and 5 dpi clearly indicate the presence of a mechanism arresting *P. viticola* growth by limiting the development of mycelium in the *Rpv32* genotype COxGT2. In contrast, the susceptible parent showed accelerated proliferation and growth of mycelium at both time points. In general, different resistance mechanisms have been studied in resistant cultivars (Bove and Rossi 2020; Wingerter et al. 2021), and the results indicated that defense reactions are triggered as soon as the haustoria are visible (Díez-Navajas et al. 2008; Yin et al. 2017). Previous studies have demonstrated that pathogen proliferation at 5 dpi clearly shows differences in spore formation of *P. viticola* between susceptible and resistant genotypes (Gindro et al. 2022; Yin et al. 2017). Significant differences in sporulation were also observed between the *V. amurensis*-derived cultivar ‘Kunbarat’ (*Rpv12*) and COxGT2 (*Rpv32*) (Fig. 5). No significant differences were observed between the wild *V. amurensis* (*Rpv8*) accession and COxGT2. The sporulation results between *Rpv8* and *Rpv12* carriers are similar to those recently reported by Müllner (2021). Detailed studies of the resistance mechanism in the resistant parent COxGT2 will provide additional information, be helpful for breeding programs, and allow the combination of resistances with different mechanisms in order to increase the degree of resistance and their durability in future varieties.

The function of leaf hairs in grapevines as a structural defense against *P. viticola* has been described in previous studies (Kortekamp and Zyprian 1999; Divilov et al. 2018). Thus, in this study, the population Gf.2018-063 was analyzed for its leaf hair density. A significant QTL for leaf hair density was identified on the upper end of chromosome 5 at 7.6 cM, which explained a phenotypic variance of up to 25.36% (Fig. 7 and Table 3). This QTL was previously also identified in the hybrid grapevine ‘Horizon’ as leaf hair locus by Divilov et al. (2017) and in ‘Muscat of Alexandria’ by Kono et al. (2018), who designated it as *Leaf Hairs 1* (*LH1*). Our QTL is mapped at (chr5_1449735 bp on chromosome 5), which overlaps but is slightly shifted from the one found by Kono et al. (2018). Therefore, we conclude that it is probably the same QTL (*LH1*). Interestingly, the detected QTL showed a contribution from the ‘Morio Muskat’ parent, which exhibited a hairless phenotype, suggesting that the locus may control the absence of leaf hairs rather than their presence, in agreement with Kono et al. (2018). In contrast to the population studied by Kono et al. (2018), which showed a clear segregation with a higher number of individuals exhibiting dense leaf hair, the present population displayed a strong skew toward low leaf hair density, with only a limited number of individuals showing high hair density. This restricted phenotypic variation limits the ability to fully assess the contribution of leaf hair density to resistance in this population. According to Kono et al. (2018), high leaf hair density was the sole factor that conferred resistance to downy mildew in their cross population. By contrast, our findings showed only a weak negative correlation between resistance to downy mildew and leaf hair density, ranging from − 0.08 (r) to − 0.12(r), indicating that leaf hair density did not significantly contribute to resistance in this population. The leaf hair density on the abaxial surface varies greatly among species (Ma et al. 2016), and some accessions of *V. labrusca* show much higher densities compared to *V. coignetiae* (Kono et al. 2018). Therefore, in this study, leaf hair density found on chromosome 5 cannot be considered an effective physical barrier to prevent infection with *P. viticola*. The observed resistance is likely determined primarily by the locus on chromosome 14. While manual evaluation was sufficient to reveal the limited contribution of leaf hair density to downy mildew resistance in this population, automated CNN-based phenotyping offers a promising avenue for more precise and scalable trichome quantification in future genetic studies (Malagol et al. 2025).

## Conclusion and future perspective

This study represents the first description of a novel resistance locus, designated as *Rpv32*, in *V. coignetiae* and the characterization of its resistance to *P. viticola*. The allele conferring resistance to downy mildew is derived from the wild species *V. coignetiae*, which has not yet been examined or exploited in grapevine breeding research. In addition to high-density rhAmpSeq markers, three SSR markers within the *Rpv32* interval are available and can be applied for MAS (Supplementary Table S1). This finding opens up new possibilities for grapevine breeders, enabling the development of new *P. viticola*-resistant varieties with new combinations of multiple resistances for environmentally friendly and sustainable viticulture. However, further investigations are necessary to elucidate the resistance mechanism for ensuring the long-term durability of future varieties and sustainable resistance management. The sequencing of the resistance donor is in progress, which will eventually shed light on the genes responsible for *Rpv32*-mediated resistance.

## Supplementary Information

Below is the link to the electronic supplementary material.

Supplementary file1 (PDF 411 KB)

Supplementary file2 (XLSX 76 KB)

Supplementary file3 (XLSX 5925 KB)

## Data Availability

All data supporting the findings of this study are available within the paper and its Supplementary Information.

## References

[CR1] Alahakoon D, Fennell A, Helget Z, Bates T, Karn A, Manns D, Mansfield AK, Reisch BI, Sacks G, Sun Q, Zou C, Cadle-Davidson L, Londo JP (2022) Berry anthocyanin, acid, and volatile trait analyses in a grapevine-interspecific F2 population using an integrated GBS and rhAmpSeq genetic map. Plants 11:696. 10.3390/plants11050696 35270166 10.3390/plants11050696 PMC8912348

[CR2] Armijo G, Schlechter R, Agurto M, Muñoz D, Nuñez C, Arce-Johnson P (2016) Grapevine pathogenic microorganisms: understanding infection strategies and host response scenarios. Front Plant Sci 7:382. 10.3389/fpls.2016.00382 27066032 10.3389/fpls.2016.00382 PMC4811896

[CR3] Barba P, Loughner R, Wentworth K, Nyrop JP, Loeb GM, Reisch BI (2019) A QTL associated with leaf trichome traits has a major influence on the abundance of the predatory mite *Typhlodromus pyri* in a hybrid grapevine population. Hortic Res 6:87. 10.1038/s41438-019-0169-8 31645947 10.1038/s41438-019-0169-8 PMC6804712

[CR4] Bellin D, Peressotti E, Merdinoglu D, Wiedemann-Merdinoglu S, Adam-Blondon AF, Cipriani G, Morgante M, Testolin R, Di Gaspero G (2009) Resistance to *Plasmopara viticola* in grapevine ‘Bianca’ is controlled by a major dominant gene causing localised necrosis at the infection site. Theor Appl Genet 120:163–176. 10.1007/s00122-009-1167-2 19821064 10.1007/s00122-009-1167-2

[CR5] Bhattarai G, Fennell A, Londo JP, Coleman C, Kovacs LG (2021) A novel grape downy mildew resistance locus from *Vitis rupestris*. Am J Enol Vitic 72:12–20

[CR6] Bitsadze N, Kikilashvili S, Chipashvili R, Mamasakhlisashvili L, Maghradze T, Kikvadze M, Toffolatti SL, De Lorenzis G, Failla O, Ocete Rubio R, Maghradze D (2024) Resistance to downy mildew in wildly growing Eurasian *Vitis vinifera* L. grapevines. J Plant Pathol 106:1759–1771. 10.1007/s42161-024-01728-7

[CR7] Blasi P, Blanc S, Wiedemann-Merdinoglu S, Prado E, Rühl EH, Mestre P, Merdinoglu D (2011) Construction of a reference linkage map of *Vitis amurensis* and genetic mapping of *Rpv8*, a locus conferring resistance to grapevine downy mildew. Theor Appl Genet 123:43–53. 10.1007/s00122-011-1565-0 21404060 10.1007/s00122-011-1565-0

[CR8] Bove F, Rossi V (2020) Components of partial resistance to *Plasmopara viticola* enable complete phenotypic characterization of grapevine varieties. Sci Rep 10:585. 10.1038/s41598-020-57482-0 31953499 10.1038/s41598-020-57482-0 PMC6969139

[CR9] Canaguier A, Grimplet J, Di Gaspero G, Scalabrin S, Duchene E, Choisne N, Mohellibi N, Guichard C, Rombauts S, Le Clainche I, Bérard A, Chauveau A, Bounon R, Rustenholz C, Morgante M, Le Paslier M-C, Brunel D, Adam-Blondon A-F (2017) A new version of the grapevine reference genome assembly (12X.v2) and of its annotation (VCost.v3). Genomics Data 14:56–62. 10.1016/j.gdata.2017.09.002 28971018 10.1016/j.gdata.2017.09.002 PMC5612791

[CR42] Core Team R (2020) R: A Language and Environment for Statistical Computing (Version 4.3.2). R Foundation for Statistical Computing. https://www.R-project.org/

[CR10] Delmotte F, Mestre P, Schneider C, Kassemeyer HH, Kozma P, Richart-Cervera S, Rouxel M, Delière L (2014) Rapid and multiregional adaptation to host partial resistance in a plant pathogenic oomycete: evidence from European populations of *Plasmopara viticola*, the causal agent of grapevine downy mildew. Infect Genet Evol 27:500–508. 10.1016/j.meegid.2013.10.017 24184095 10.1016/j.meegid.2013.10.017

[CR11] Diez-Navajas A, Wiedemann-Merdinoglu S, Greif C, Merdinoglu D (2008) Nonhost versus host resistance to the grapevine downy mildew, *Plasmopara viticola*, studied at the tissue level. Phytopathology 98:776–780. 10.1094/PHYTO-98-7-0776 18943253 10.1094/PHYTO-98-7-0776

[CR12] Divilov K, Wiesner-Hanks T, Barba P, Cadle-Davidson L, Reisch BI (2017) Computer vision for high-throughput quantitative phenotyping: a case study of grapevine downy mildew sporulation and leaf trichomes. Phytopathology 107:1549–1555. 10.1094/PHYTO-04-17-0137-R 28745103 10.1094/PHYTO-04-17-0137-R

[CR13] Divilov K, Barba P, Cadle-Davidson L, Reisch RI (2018) Single and multiple phenotype QTL analyses of downy mildew resistance in interspecific grapevines. Theor Appl Genet 131:1133–1143. 10.1007/s00122-018-3065-y 29417162 10.1007/s00122-018-3065-y PMC5895686

[CR14] Droulia F, Charalampopoulos I (2021) Future climate change impacts on European viticulture: a review on recent scientific advances. Atmosphere 12:495. 10.3390/atmos12040495

[CR15] Eibach R, Töpfer R (2015) Traditional grapevine breeding techniques. In: Reynolds A (ed) Grapevine breeding programs for the wine industry. Woodhead Publishing, Oxford, United Kingdom, pp 3–22. 10.1016/B978-1-78242-075-0.00001-6

[CR16] Eibach R, Zyprian E, Welter L, Töpfer R (2007) The use of molecular markers for pyramiding resistance genes in grapevine breeding. Vitis 46:120–124. 10.5073/vitis.2007.46.120-124

[CR17] Eisenmann B, Czemmel S, Ziegler T, Buchholz G, Kortekamp A, Trapp O, Rausch T, Dry I, Bogs J (2019) *Rpv3-1* mediated resistance to grapevine downy mildew is associated with specific host transcriptional responses and the accumulation of stilbenes. BMC Plant Biol 19:1–17. 10.1186/s12870-019-1935-3 31387524 10.1186/s12870-019-1935-3 PMC6685164

[CR18] Eisenmann B, Wingerter C, Dressler M, Freund C, Kortekamp A, Bogs J (2023) Fungicide-saving potential and economic advantages of fungus-resistant grapevine cultivars. Plants 12:3120. 10.3390/plants12173120 37687364 10.3390/plants12173120 PMC10489737

[CR19] Fu P, Wu W, Lai G, Li R, Peng Y, Yang B, Wang B, Yin L, Qu J, Song S, Lu J (2020) Identifying *Plasmopara viticola* resistance loci in grapevine (*Vitis amurensis*) via genotyping-by-sequencing-based QTL mapping. Plant Physiol Biochem 154:75–84. 10.1016/j.plaphy.2020.05.016 32535323 10.1016/j.plaphy.2020.05.016

[CR20] Gago P, Conejero G, Martínez MC, Boso S, This P, Verdeil JL (2016) Microanatomy of leaf trichomes: opportunities for improved ampelographic discrimination of grapevine (*Vitis vinifera* L.) cultivars. Austral J Grape Wine Res 22:494–503. 10.1111/ajgw.12226

[CR21] Galet P (1988) Cépages et vignobles de France. Tome I: Les vignes américaines, 2ème édition. Imprimerie Charles Dehan, Montpellier, France

[CR22] Gessler C, Pertot I, Perazzolli M (2011) *Plasmopara viticola*: a review of knowledge on downy mildew of grapevine and effective disease management. Phytopathol Mediterr 50:3–44. 10.14601/Phytopathol_Mediterr-9360

[CR23] Gindro K, Schnee S, Lecoultre N, Michellod E, Zufferey V, Spring JL, Viret O, Dubuis PH (2022) Development of downy mildew in grape bunches of susceptible and resistant cultivars: infection pathways and limited systemic spread. Austral J Grape Wine Res 28:572–580. 10.1111/ajgw.12560

[CR24] Karn A, Diaz-Garcia L, Reshef N, Zou C, Manns DC, Cadle-Davidson L, Mansfield AK, Reisch BI, Sacks GL (2021) The genetic basis of anthocyanin acylation in North American grapes (*Vitis spp.*). Genes 12:1962. 10.3390/genes12121962 34946911 10.3390/genes12121962 PMC8701791

[CR25] Kassemeyer H-H, Gadoury DM, Hill G, Wilcox WF (2015) Downy mildew. In: Wilcox WF, Gubler WD, Uyemoto JK (eds) Compendium of grape diseases, disorders, and pests, 2nd edn. APS Press, St. Paul, MN, USA, pp 46–52. 10.1094/9780890544815

[CR26] Koledenkova K, Esmaeel Q, Jacquard C, Nowak J, Clément C, Barka EA (2022) *Plasmopara viticola* the causal agent of downy mildew of grapevine: from its taxonomy to disease management. Front Microbiol 13:889472. 10.3389/fmicb.2022.889472 35633680 10.3389/fmicb.2022.889472 PMC9130769

[CR27] Kono A, Ban Y, Mitani N, Fujii H, Sato S, Suzaki K, Azuma A, Onoue N, Sato A (2018) Development of SSR markers linked to QTL reducing leaf hair density and grapevine downy mildew resistance in *Vitis vinifera*. Mol Breed 38:138. 10.1007/s11032-018-0889-8

[CR28] Kortekamp A, Zyprian E (1999) Leaf hairs as a basic protective barrier against downy mildew of grape. J Phytopathol 147:453–459. 10.1111/j.1439-0434.1999.tb03850.x

[CR29] Kortekamp A, Wind R, Zyprian E (1999) The role of hairs on the wettability of grapevine (*Vitis* spp.) leaves. Vitis 38:101–106. 10.5073/vitis.1999.38.101-105

[CR30] Lin H, Leng H, Guo Y, Kondo S, Zhao Y, Shi G, Guo X (2019) QTLs and candidate genes for downy mildew resistance conferred by interspecific grape (*V. vinifera* L. × *V. amurensis* Rupr.) crossing. Sci Hortic 244:200–207. 10.1016/j.scienta.2018.09.045

[CR31] Ma ZY, Wen J, Ickert-Bond SM, Chen LQ, Liu XQ (2016) Morphology, structure, and ontogeny of trichomes of the grape genus (*Vitis*, Vitaceae). Front Plant Sci 7:704. 10.3389/fpls.2016.00704 27252720 10.3389/fpls.2016.00704 PMC4879774

[CR32] Malagol N, Rao T, Werner A, Töpfer R, Hausmann L (2025) A high-throughput ResNet CNN approach for automated grapevine leaf hair quantification. Sci Rep 15:1590. 10.1038/s41598-025-85336-0 39794464 10.1038/s41598-025-85336-0 PMC11724064

[CR33] Marguerit E, Boury C, Manicki A, Donnart M, Butterlin G, Némorin A, Wiedemann-Merdinoglu S, Merdinoglu D, Ollat N, Decroocq S (2009) Genetic dissection of sex determinism, inflorescence morphology and downy mildew resistance in grapevine. Theor Appl Genet 118:1261–1278. 10.1007/s00122-009-0979-4 19238349 10.1007/s00122-009-0979-4

[CR34] Marinho MdaC, Diogo BS, Lage OM, Antunes SC (2020) Ecotoxicological evaluation of fungicides used in viticulture in non-target organisms. Environ Sci Pollut Res 27:43958–43969. 10.1007/s11356-020-10245-w 10.1007/s11356-020-10245-w 32748361

[CR35] Massi F, Torriani SFF, Borghi L, Toffolatti SL (2021) Fungicide resistance evolution and detection in plant pathogens: *Plasmopara viticola* as a case study. Microorganisms 9:119. 10.3390/microorganisms9010119 33419171 10.3390/microorganisms9010119 PMC7825580

[CR36] Merdinoglu D, Schneider C, Prado E, Wiedemann-Merdinoglu S, Mestre P (2018) Breeding for durable resistance to downy and powdery mildew in grapevine. OENO One 52:203–209. 10.20870/oeno-one.2018.52.3.2116

[CR37] Müllner S (2021) Zytologische und molekulare Studien zum Resistenzlocus *Rpv12* gegen den Falschen Mehltau der Rebe (*Plasmopara viticola*). Dissertation, Julius Kühn-Institut. 10.5073/20211105-095339

[CR38] OIV (2009) OIV descriptor list for grape varieties and *Vitis* species, 2nd edn. Organization Internationale de la Vigne et du Vin, Paris

[CR39] Park M, Vera D, Kambrianda D, Gajjar P, Cadle-Davidson L, Tsolova V, El-Sharkawy I (2022) Chromosome-level genome sequence assembly and genome-wide association study of *Muscadinia rotundifolia* reveal the genetics of 12 berry-related traits. Hortic Res 9:uhab011. 10.1093/hr/uhab011 35040982 10.1093/hr/uhab011 PMC8769032

[CR40] Peressotti E, Wiedemann-Merdinoglu S, Delmotte F, Bellin D, Di Gaspero G, Testolin R, Merdinoglu D, Mestre P (2010) Breakdown of resistance to grapevine downy mildew upon limited deployment of a resistant variety. BMC Plant Biol 10:147. 10.1186/1471-2229-10-147 20633270 10.1186/1471-2229-10-147 PMC3095292

[CR41] Petucco C, Roderich M-S, Molitor D, Heilemann K, Simon C, Rugani B, Beyer M (2026) Fungus-resistant grape cultivars have up to threefold lower environmental impacts than traditional varieties. Sci Total Environ 1027:181700. 10.1016/j.scitotenv.2026.181700 41875509 10.1016/j.scitotenv.2026.181700

[CR43] Rastas P (2017) Lep-MAP3: robust linkage mapping even for low-coverage whole genome sequencing data. Bioinformatics 33:3726–3732. 10.1093/bioinformatics/btx494 29036272 10.1093/bioinformatics/btx494

[CR44] Reisch BI, Cadle-Davidson L, Ikeogu U, Sacks GL, Londo JP, Martinson TE (2023) Contributions of the VitisGen2 project to grapevine breeding and genetics. Vitis 62:88–91. 10.5073/vitis.2023.62.special-issue.88-91

[CR45] Reshef N, Karn A, Manns DC, Mansfield AK, Cadle-Davidson L, Reisch B, Sacks GL (2022) Stable QTL for malate levels in ripe fruit and their transferability across *Vitis* species. Hortic Res 9:uhac009. 10.1093/hr/uhac009 35369130 10.1093/hr/uhac009 PMC8968676

[CR46] Sánchez-Mora FD, Saifert L, Zanghelini J, Paixão CA, Dal Vesco LL, Eibach R, Dalbó MA, Nodari RO, Welter LJ (2022) Pyramiding of resistance alleles to grape powdery mildew assisted by molecular markers. Crop Breed Appl Biotech 22:e42252247. 10.1590/1984-70332022v22n4a42

[CR48] Sapkota S, Chen L-L, Yang S, Hyma KE, Cadle-Davidson L, Hwang C-F (2019) Construction of a high-density linkage map and QTL detection of downy mildew resistance in *Vitis aestivalis*-derived ‘Norton.’ Theor Appl Genet 132:137–147. 10.1007/s00122-018-3203-6 30341491 10.1007/s00122-018-3203-6

[CR49] Sapkota S, Zou C, Ledbetter C, Underhill A, Sun Q, Gadoury D, Cadle-Davidson L (2023) Discovery and genome-guided mapping of *REN12* from *Vitis amurensis*, conferring strong, rapid resistance to grapevine powdery mildew. Hortic Res 10:uhad052. 10.1093/hr/uhad052 37213681 10.1093/hr/uhad052 PMC10194894

[CR50] Sargolzaei M, Maddalena G, Bitsadze N, Maghradze D, Bianco PA, Failla O, Toffolatti SL, De Lorenzis G (2020) *Rpv29*, *Rpv30* and *Rpv31*: three novel genomic loci associated with resistance to *Plasmopara viticola* in *Vitis vinifera*. Front Plant Sci 11:562432. 10.3389/fpls.2020.562432 33163011 10.3389/fpls.2020.562432 PMC7583455

[CR51] Schneider C, Onimus C, Prado E, Dumas V, Wiedemann-Merdinoglu S, Dorne MA, Lacombe MC, Piron MC, Umar-Faruk A, Duchene E, Mestre P, Merdinoglu D (2019) INRA-ResDur: the French grapevine breeding programme for durable resistance to downy and powdery mildew. Acta Hortic 1248:207–213. 10.17660/ActaHortic.2019.1248.30

[CR52] Schwander F, Eibach R, Fechter I, Hausmann L, Zyprian E, Toepfer R (2012) Rpv10: a new locus from the Asian *Vitis* gene pool for pyramiding downy mildew resistance loci in grapevine. Theor Appl Genet 124:163–176. 10.1007/s00122-011-1695-4 21935694 10.1007/s00122-011-1695-4

[CR53] Scott ES, Dambergs RG, Stummer BE, Petrovic T (2022) Fungal contaminants in the vineyard and wine quality and safety. In: Reynolds A (ed) Managing wine quality, 2nd edn. Woodhead Publishing, Oxford, United Kingdom, pp 587–623. 10.1016/B978-0-08-102067-8.00006-3

[CR54] Tomaz I, Buljević N, Šikuten I, Preiner D (2025) Advancing grapevine breeding with reliable SSR genotyping: the Qsep100 approach. Horticulturae 11:1506. 10.3390/horticulturae11121506

[CR55] Töpfer R, Hausmann L, Harst M, Maul E, Zyprian E, Eibach R (2011) New horizons for grapevine breeding. In: Flachowsky H, Hanke MV (eds) Methods in Temperate Fruit Breeding. Fruit, vegetable and cereal science and biotechnology 5:79–100. http://www.globalsciencebooks.info/Online/GSBOnline/images/2011/FVCSB_5(SI1)/FVCSB_5(SI1)79-100o.pdf

[CR56] Töpfer R, Trapp O (2022) A cool climate perspective on grapevine breeding: climate change and sustainability are driving forces for changing varieties in a traditional market. Theor Appl Genet 135:3947–3960. 10.1007/s00122-022-04077-0 35389053 10.1007/s00122-022-04077-0 PMC9729149

[CR57] Trapp O, Avia K, Borrelli C, Eibach R, Merdinoglu D, Töpfer R (2025) More sustainability in Europe’s vineyards – using resistant grapevine varieties to reduce the input of pesticides. Plants People Planet 7:1621–1628. 10.1002/ppp3.70038

[CR58] van Loon LC, Rep M, Pieterse CMJ (2006) Significance of inducible defense-related proteins in infected plants. Annu Rev Phytopathol 44:135–162. 10.1146/annurev.phyto.44.070505.143425 16602946 10.1146/annurev.phyto.44.070505.143425

[CR59] Venuti S, Copetti D, Foria S, Falginella L, Hoffmann S, Bellin D, Cindric P, Kozma P, Scalabrin S, Morgante M, Testolin R, Di Gaspero G (2013) Historical introgression of the downy mildew resistance gene *Rpv12* from the Asian species *Vitis amurensis* into grapevine varieties. PLoS One. 10.1371/journal.pone.0061228 23593440 10.1371/journal.pone.0061228 PMC3625174

[CR60] Vezzulli S, Gramaje D, Tello J, Gambino G, Bettinelli P, Pirrello C, Schwandner A, Barba P, Angelini E, Anfora GF, Mazzoni V, Pozzebon A, Palomares-Rius JE, Martínez-Diz MP, Toffolatti SL, De Lorenzis G, De Paoli E, Perrone I, D’Incà E, Zenoni S, Wilmink J, Lacombe T, Crespan M, Walker MA, Bavaresco L, De la Fuente M, Fennel A, Tornielli GB, Forneck A, Ibañez J, Hausmann L, Reisch IB (2022) Genomic designing for biotic stress resistant grapevine. In: Kole C (ed) Genomic designing for biotic stress resistant fruit crops. Springer, Cham Switzerland, pp 87–255. 10.1007/978-3-030-91802-6_4

[CR61] Wagner GJ, Wang E, Shepherd R (2004) New approaches for studying and exploiting an old protuberance, the plant trichome. Ann Bot 93:3–11. 10.1093/aob/mch011 14678941 10.1093/aob/mch011 PMC4242265

[CR62] Welter LJ, Göktürk-Baydar N, Akkurt M, Maul E, Eibach R, Töpfer R, Zyprian E (2007) Genetic mapping and localization of quantitative trait loci affecting fungal disease resistance and leaf morphology in grapevine (*Vitis vinifera* L). Mol Breed 20:359–374. 10.1007/s11032-007-9097-7

[CR63] Wightwick A, Walters R, Allinson G, Reichman S, Menzies N (2010) Environmental risks of fungicides used in horticultural production systems. In: C O (ed) Fungicides. InTech, Rijeka, Croatia, pp 273–304

[CR64] Wingerter C, Eisenmann B, Weber P, Dry I, Bogs J (2021) Grapevine *Rpv3*-, *Rpv10*- and *Rpv12*-mediated defense responses against *Plasmopara viticola* and the impact of their deployment on fungicide use in viticulture. BMC Plant Biol 21:470. 10.1186/s12870-021-03228-7 34649524 10.1186/s12870-021-03228-7 PMC8515710

[CR65] Yin X, Liu RQ, Su H, Su L, Guo YR, Wang ZJ, Du W, Li M-J, Zhang X, Wang Y-J, Liu G-T, Xu Y (2017) Pathogen development and host responses to *Plasmopara viticola* in resistant and susceptible grapevines: an ultrastructural study. Hortic Res 4:17033. 10.1038/hortres.2017.33 28785414 10.1038/hortres.2017.33 PMC5539432

[CR66] Yin L, Karn A, Cadle-Davidson L, Zou C, Underhill A, Atkins P, Treiber E, Voytas D, Clark M (2021) Fine mapping of leaf trichome density revealed a 747-kb region on chromosome 1 in cold-hardy hybrid wine grape populations. Front Plant Sci 12:587640. 10.3389/fpls.2021.587640 33746993 10.3389/fpls.2021.587640 PMC7965957

[CR67] Zendler D, Malagol N, Schwandner A, Töpfer R, Hausmann L, Zyprian E (2021) High-throughput phenotyping of leaf discs infected with grapevine downy mildew using shallow convolutional neural networks. Agronomy 11:1768. 10.3390/agronomy11091768

[CR68] Zini E, Dolzani C, Stefanini M, Gratl V, Bettinelli P, Nicolini D, Betta G, Dorigatti C, Velasco R, Letschka T, Vezzulli S (2019) *R*-loci arrangement versus downy and powdery mildew resistance level: a *Vitis* hybrid survey. Int J Mol Sci 20:3526. 10.3390/ijms20143526 31323823 10.3390/ijms20143526 PMC6679420

[CR69] Zou C, Karn A, Reisch B, Nguyen A, Sun Y, Bao Y, Campbell MS, Church D, Williams S, Xu X, Ledbetter CA, Patel S, Fennell A, Glaubitz JC, Clark M, Ware D, Londo JP, Sun Q, Cadle-Davidson L (2020) Haplotyping the *Vitis* collinear core genome with rhAmpSeq improves marker transferability in a diverse genus. Nat Commun 11:413. 10.1038/s41467-019-14280-1 31964885 10.1038/s41467-019-14280-1 PMC6972940

[CR70] Zou C, Sapkota S, Figueroa-Balderas R, Glaubitz J, Cantu D, Kingham BF, Sun Q, Cadle-Davidson L (2023) A multitiered haplotype strategy to enhance phased assembly and fine mapping of a disease resistance locus. Plant Physiol 193:2321–2336. 10.1093/plphys/kiad494 37706526 10.1093/plphys/kiad494

