# Supplementary material for: A novel resistance locus from an unexploited East Asian Vitis coignetiae confers resistance to grapevine downy mildew
Source: Theor Appl Genet. 2026 Jun 1;139(6):170. doi: 10.1007/s00122-026-05273-y (PMC13226344; doi:10.1007/s00122-026-05273-y)
Supplement: Supplementary file 1 — Supplementary file1 (PDF 411 KB) [file 122_2026_5273_MOESM1_ESM.pdf]

Supplementary Information

This supplementary file1 contains 1 table and 4 figures.

A novel resistance locus from an unexploited East Asian *Vitis coignetiae* confers resistance to grapevine downy mildew

Nagarjun Malagol, Anna Werner, Reinhard Töpfer, Ludger Hausmann*

Theoretical and Applied Genetics (2026)

Julius Kuehn Institute (JKI), Federal Research Centre for Cultivated Plants, Institute for Grapevine Breeding, Geilweilerhof, 76833 Siebeldingen, Germany

* Corresponding author: ludger.hausmann@julius-kuehn.de

Table S1. Allele sizes of SSR markers associated with the *Rpv32* locus. VMC1E12 is close to the LODmax marker chr14_6974992, UDV-057 and UDV-336 are flanking markers. Haplotype #2 of rhAmpSeq marker chr5_1449735 associated with hairlessness of 'Morio Muskat'.

<table>
<tr><th>Marker</th><th>Chr</th><th>Physical position [bp]</th><th>Forward primer</th><th>Reverse primer</th><th>Fluo label</th><th>COxGT2
Allele size [bp]</th></tr>
<tr><td>UDV-057</td><td>14</td><td>4814318</td><td>CAGGCAAGGTTCACAACAGA</td><td>TGTGAGCCTCTCCTCTTCGT</td><td>HEX</td><td>176/183</td></tr>
<tr><td>VMC1E12</td><td>14</td><td>7136672</td><td>GTGTGACCTTATGCAACACCAA</td><td>GCTACCACATGCAGACAGGTTAGT</td><td>HEX</td><td>238/256</td></tr>
<tr><td>UDV-336</td><td>14</td><td>7908061</td><td>GCCCAAGGGTTCTTTATGTTC</td><td>TCAATTGAGGTCTTAACCAATGAA</td><td>6-FAM</td><td>161/210</td></tr>
<tr><th>Marker</th><th>Chr</th><th>Physical position [bp]</th><th colspan="4">Allele sequence of 'Morio Muskat'</th></tr>
<tr><td>chr5_1449735#2</td><td>5</td><td>1449735</td><td colspan="4">GTGAGCACATCAGACAATTTCCTATTTTCCTTTACATATGATGATTTAGGCCCTACTTTCCTTACACAAGAAGCTAGAATG
TTAAAAATGAAAGATTCTTTGTGCTTACTATCAACATGGAATTATGGAATTATATATATATACATACATACATACATTCAA
CAATAAAATTAAAATGATAATAAATGAACAAAGTCAAACACTAACC</td></tr>
</table>

The physical position is based on the reference genome PN40024 12X.v2 (Canaguier et al. 2017). Alleles associated with resistance to downy mildew from COxGT2 are highlighted in bold.

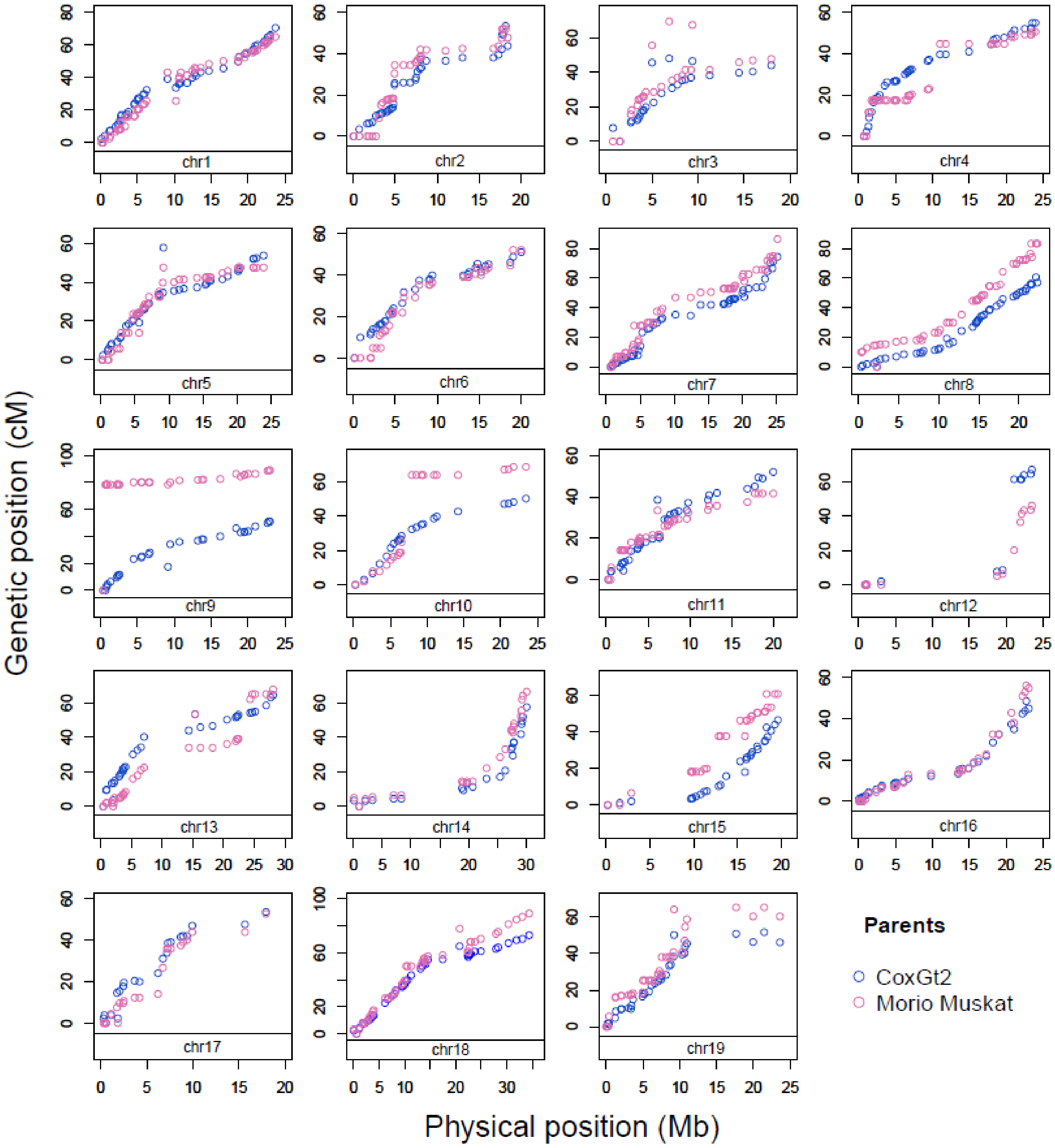

Supplementary Figure S1. Collinearity between genetic and physical marker positions across the 19 grapevine chromosomes in the 'Morio Muskat' × COxGT2 mapping population Gf.2018-063. Scatter plots show the relationship between genetic positions (y-axis: cM) and physical positions (x-axis: Mb) of rhAmpSeq markers for each chromosome. Blue circles represent the resistant parent COxGT2, and pink circles represent the susceptible parent 'Morio Muskat'.

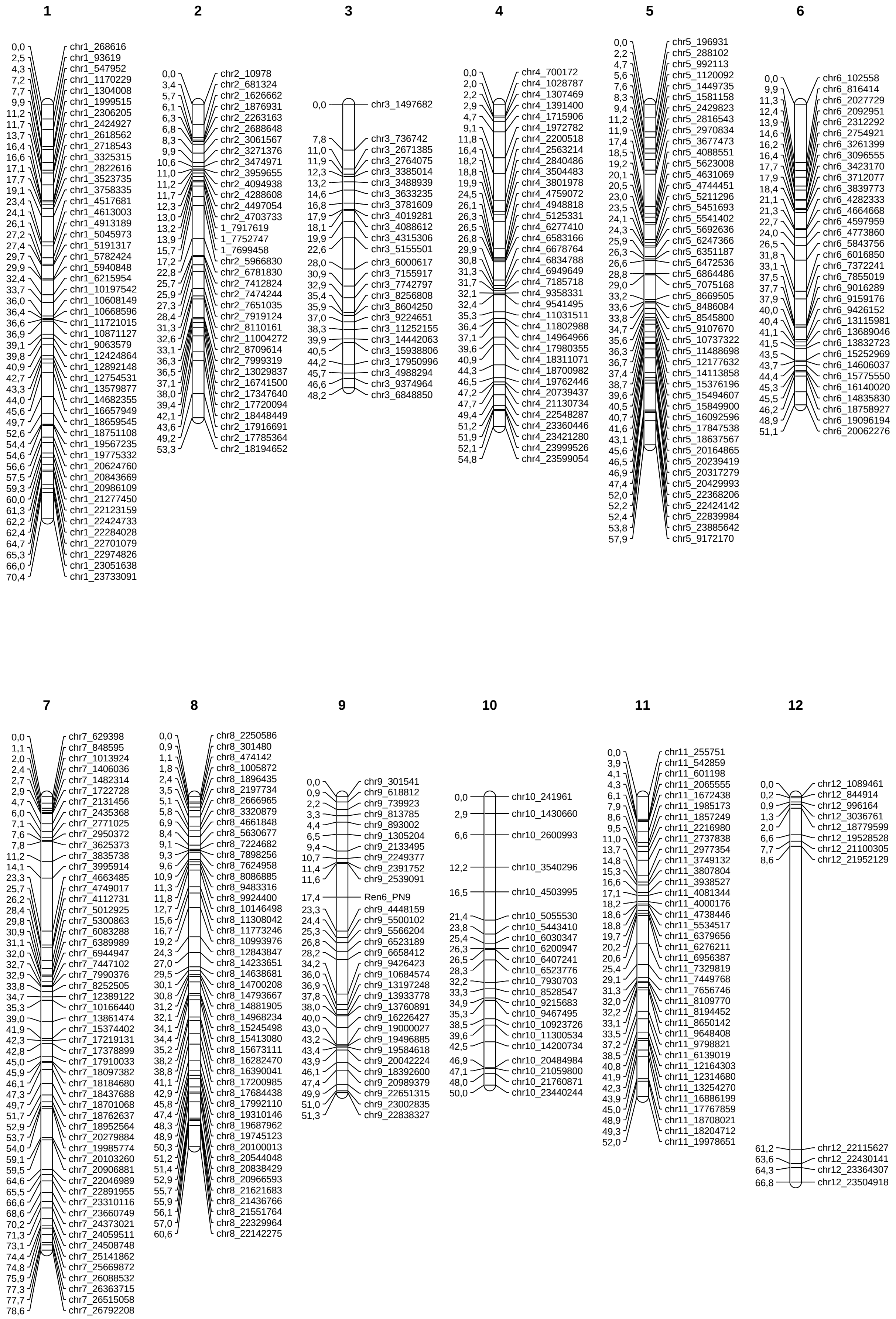
1
0,0 chr1_268616
2,5 chr1_93619
4,3 chr1_547952
7,2 chr1_1170229
7,7 chr1_1304008
9,9 chr1_1999515
11,2 chr1_2306205
11,7 chr1_2424927
13,7 chr1_2618562
16,4 chr1_2718543
16,6 chr1_3325315
17,1 chr1_2822616
17,7 chr1_3523735
19,1 chr1_3758335
23,4 chr1_4517681
24,1 chr1_4613003
26,1 chr1_4913189
27,2 chr1_5045973
27,4 chr1_5191317
29,7 chr1_5782424
29,9 chr1_5940848
32,4 chr1_6215954
33,7 chr1_10197542
36,0 chr1_10608149
36,4 chr1_10668596
36,6 chr1_11721015
36,9 chr1_10871127
39,1 chr1_9063579
39,8 chr1_12424864
40,9 chr1_12892148
42,7 chr1_12754531
43,3 chr1_13579877
44,0 chr1_14682355
45,6 chr1_16657949
49,7 chr1_18659545
52,6 chr1_18751108
54,4 chr1_19567235
54,6 chr1_19775332
56,6 chr1_20624760
57,5 chr1_20843669
59,3 chr1_20986109
60,0 chr1_21277450
61,3 chr1_22123159
62,2 chr1_22424733
62,4 chr1_22284028
64,7 chr1_22701079
65,3 chr1_22974826
66,0 chr1_23051638
70,4 chr1_23733091
2
0,0 chr2_10978
3,4 chr2_681324
5,7 chr2_1626662
6,1 chr2_1876931
6,3 chr2_2263163
6,8 chr2_2688648
8,3 chr2_3061567
9,9 chr2_3271376
10,6 chr2_3474971
11,0 chr2_3959655
11,2 chr2_4094938
11,7 chr2_4288608
12,3 chr2_4497054
13,0 chr2_4703733
13,2 1_7917619
13,9 1_7752747
15,7 1_7699458
17,2 chr2_5966830
22,8 chr2_6781830
25,7 chr2_7412824
25,9 chr2_7474244
27,3 chr2_7651035
28,4 chr2_7919124
31,3 chr2_8110161
32,6 chr2_11004272
33,1 chr2_8709614
36,3 chr2_7999319
36,5 chr2_13029837
37,1 chr2_16741500
38,0 chr2_17347640
39,4 chr2_17720094
42,1 chr2_18448449
43,6 chr2_17916691
49,2 chr2_17785364
53,3 chr2_18194652
3
0,0 chr3_1497682
7,8 chr3_736742
11,0 chr3_2671385
11,9 chr3_2764075
12,3 chr3_3385014
13,2 chr3_3488939
14,6 chr3_3633235
16,8 chr3_3781609
17,9 chr3_4019281
18,1 chr3_4088612
19,9 chr3_4315306
22,6 chr3_5155501
28,0 chr3_6000617
30,9 chr3_7155917
32,9 chr3_7742797
35,4 chr3_8256808
35,9 chr3_8604250
37,0 chr3_9224651
38,3 chr3_11252155
39,9 chr3_14442063
40,5 chr3_15938806
44,2 chr3_17950996
45,7 chr3_4988294
46,6 chr3_9374964
48,2 chr3_6848850
4
0,0 chr4_700172
2,0 chr4_1028787
2,2 chr4_1307469
2,9 chr4_1391400
4,7 chr4_1715906
9,1 chr4_1972782
11,8 chr4_2200518
16,4 chr4_2563214
18,2 chr4_2840486
18,8 chr4_3504483
19,9 chr4_3801978
24,5 chr4_4759072
26,1 chr4_4948818
26,3 chr4_5125331
26,5 chr4_6277410
26,8 chr4_6583166
29,9 chr4_6678764
30,8 chr4_6834788
31,3 chr4_6949649
31,7 chr4_7185718
32,1 chr4_9358331
32,4 chr4_9541495
35,3 chr4_11031511
36,4 chr4_11802988
37,1 chr4_14964966
39,6 chr4_17980355
40,9 chr4_18311071
44,3 chr4_18700982
46,5 chr4_19762446
47,2 chr4_20739437
47,7 chr4_21130734
49,4 chr4_22548287
51,2 chr4_23360446
51,9 chr4_23421280
52,1 chr4_23999526
54,8 chr4_23599054
5
0,0 chr5_196931
2,2 chr5_288102
4,7 chr5_992113
5,6 chr5_1120092
7,6 chr5_1449735
8,3 chr5_1581158
9,4 chr5_2429823
11,2 chr5_2816543
11,9 chr5_2970834
17,4 chr5_3677473
18,5 chr5_4088551
19,2 chr5_5623008
20,1 chr5_4631069
20,5 chr5_4744451
23,0 chr5_5211296
23,5 chr5_5451693
24,1 chr5_5541402
24,3 chr5_5692636
25,9 chr5_6247366
26,3 chr5_6351187
26,6 chr5_6472536
28,8 chr5_6864486
29,0 chr5_7075168
33,2 chr5_8669505
33,6 chr5_8486084
33,8 chr5_8545800
34,7 chr5_9107670
35,6 chr5_10737322
36,3 chr5_11488698
36,7 chr5_12177632
37,4 chr5_14113858
38,7 chr5_15376196
39,6 chr5_15494607
40,5 chr5_15849900
40,7 chr5_16092596
41,6 chr5_17847538
43,1 chr5_18637567
45,6 chr5_20164865
46,5 chr5_20239419
46,9 chr5_20317279
47,4 chr5_20429993
52,0 chr5_22368206
52,2 chr5_22424142
52,4 chr5_22839984
53,8 chr5_23885642
57,9 chr5_9172170
6
0,0 chr6_102558
9,9 chr6_816414
11,3 chr6_2027729
12,4 chr6_2092951
13,9 chr6_2312292
14,6 chr6_2754921
16,2 chr6_3261399
16,4 chr6_3096555
17,7 chr6_3423170
17,9 chr6_3712077
18,4 chr6_3839773
21,1 chr6_4282333
21,3 chr6_4664668
22,7 chr6_4597959
24,0 chr6_4773860
26,5 chr6_5843756
31,8 chr6_6016850
33,1 chr6_7372241
37,5 chr6_7855019
37,7 chr6_9016289
37,9 chr6_9159176
40,0 chr6_9426152
40,4 chr6_13115981
41,1 chr6_13689046
41,5 chr6_13832723
43,5 chr6_15252969
43,7 chr6_14606037
44,4 chr6_15775550
45,3 chr6_16140020
45,5 chr6_14835830
46,2 chr6_18758927
48,9 chr6_19096194
51,1 chr6_20062276
7
0,0 chr7_629398
1,1 chr7_848595
2,0 chr7_1013924
2,4 chr7_1406036
2,7 chr7_1482314
2,9 chr7_1722728
4,7 chr7_2131456
6,0 chr7_2435368
7,1 chr7_2771025
7,6 chr7_2950372
7,8 chr7_3625373
11,2 chr7_3835738
14,1 chr7_3995914
23,3 chr7_4663485
25,7 chr7_4749017
26,2 chr7_4112731
28,4 chr7_5012925
29,8 chr7_5300863
30,9 chr7_6083288
31,1 chr7_6389989
32,0 chr7_6944947
32,7 chr7_7447102
32,9 chr7_7990376
33,8 chr7_8252505
34,7 chr7_12389122
35,3 chr7_10166440
39,0 chr7_13861474
41,9 chr7_15374402
42,3 chr7_17219131
42,8 chr7_17378899
45,0 chr7_17910033
45,9 chr7_18097382
46,1 chr7_18184680
47,3 chr7_18437688
49,7 chr7_18701068
51,7 chr7_18762637
52,9 chr7_18952564
53,7 chr7_20279884
54,0 chr7_19985774
59,1 chr7_20103260
59,5 chr7_20906881
64,6 chr7_22046989
65,5 chr7_22891955
66,6 chr7_23310116
68,6 chr7_23660749
70,2 chr7_24373021
71,3 chr7_24059511
73,1 chr7_24508748
74,4 chr7_25141862
74,8 chr7_25669872
75,9 chr7_26088532
77,3 chr7_26363715
77,7 chr7_26515058
78,6 chr7_26792208
8
0,0 chr8_2250586
0,9 chr8_301480
1,1 chr8_474142
1,8 chr8_1005872
2,4 chr8_1896435
3,5 chr8_2197734
5,1 chr8_2666965
5,8 chr8_3320879
6,9 chr8_4661848
8,4 chr8_5630677
9,1 chr8_7224682
9,3 chr8_7898256
9,6 chr8_7624958
10,9 chr8_8086885
11,3 chr8_9483316
11,8 chr8_9924400
12,7 chr8_10146498
15,6 chr8_11308042
16,7 chr8_11773246
19,2 chr8_10993976
24,3 chr8_12843847
27,0 chr8_14233651
29,5 chr8_14638681
30,1 chr8_14700208
30,8 chr8_14793667
31,2 chr8_14881905
32,1 chr8_14968234
34,1 chr8_15245498
34,4 chr8_15413080
35,2 chr8_15673111
38,2 chr8_16282470
38,8 chr8_16390041
41,1 chr8_17200985
42,9 chr8_17684438
45,8 chr8_17992110
47,4 chr8_19310146
48,3 chr8_19687962
48,9 chr8_19745123
50,3 chr8_20100013
51,2 chr8_20544048
51,4 chr8_20838429
52,9 chr8_20966593
55,7 chr8_21621683
55,9 chr8_21436766
56,1 chr8_21551764
57,0 chr8_22329964
60,6 chr8_22142275
9
0,0 chr9_301541
0,9 chr9_618812
2,2 chr9_739923
3,3 chr9_813785
4,4 chr9_893002
6,5 chr9_1305204
9,4 chr9_2133495
10,7 chr9_2249377
11,4 chr9_2391752
11,6 chr9_2539091
17,4 Ren6_PN9
23,3 chr9_4448159
24,4 chr9_5500102
25,3 chr9_5566204
26,8 chr9_6523189
28,2 chr9_6658412
34,2 chr9_9426423
36,0 chr9_10684574
36,9 chr9_13197248
37,8 chr9_13933778
38,0 chr9_13760891
40,0 chr9_16226427
43,0 chr9_19000027
43,2 chr9_19496885
43,4 chr9_19584618
43,9 chr9_20042224
46,1 chr9_18392600
47,4 chr9_20989379
49,9 chr9_22651315
51,0 chr9_23002835
51,3 chr9_22838327
10
0,0 chr10_241961
2,9 chr10_1430660
6,6 chr10_2600993
12,2 chr10_3540296
16,5 chr10_4503995
21,4 chr10_5055530
23,8 chr10_5443410
25,4 chr10_6030347
26,3 chr10_6200947
26,5 chr10_6407241
28,3 chr10_6523776
32,2 chr10_7930703
33,3 chr10_8528547
34,9 chr10_9215683
35,3 chr10_9467495
38,5 chr10_10923726
39,6 chr10_11300534
42,5 chr10_14200734
46,9 chr10_20484984
47,1 chr10_21059800
48,0 chr10_21760871
50,0 chr10_23440244
11
0,0 chr11_255751
3,9 chr11_542859
4,1 chr11_601198
4,3 chr11_2065555
6,1 chr11_1672438
7,9 chr11_1985173
8,6 chr11_1857249
9,5 chr11_2216980
11,0 chr11_2737838
13,7 chr11_2977354
14,8 chr11_3749132
15,3 chr11_3807804
16,6 chr11_3938527
17,1 chr11_4081344
18,2 chr11_4000176
18,6 chr11_4738446
18,8 chr11_5534517
19,7 chr11_6379656
20,2 chr11_6276211
20,6 chr11_6956387
25,4 chr11_7329819
29,1 chr11_7449768
31,3 chr11_7656746
32,0 chr11_8109770
32,2 chr11_8194452
33,1 chr11_8650142
33,5 chr11_9648408
37,2 chr11_9798821
38,5 chr11_6139019
40,8 chr11_12164303
41,9 chr11_12314680
42,3 chr11_13254270
43,9 chr11_16886199
45,0 chr11_17767859
48,9 chr11_18708021
49,3 chr11_18204712
52,0 chr11_19978651
12
0,0 chr12_1089461
0,2 chr12_844914
0,9 chr12_996164
1,3 chr12_3036761
2,0 chr12_18779599
6,6 chr12_19528528
7,7 chr12_21100305
8,6 chr12_21952129
61,2 chr12_22115627
63,6 chr12_22430141
64,3 chr12_23364307
66,8 chr12_23504918

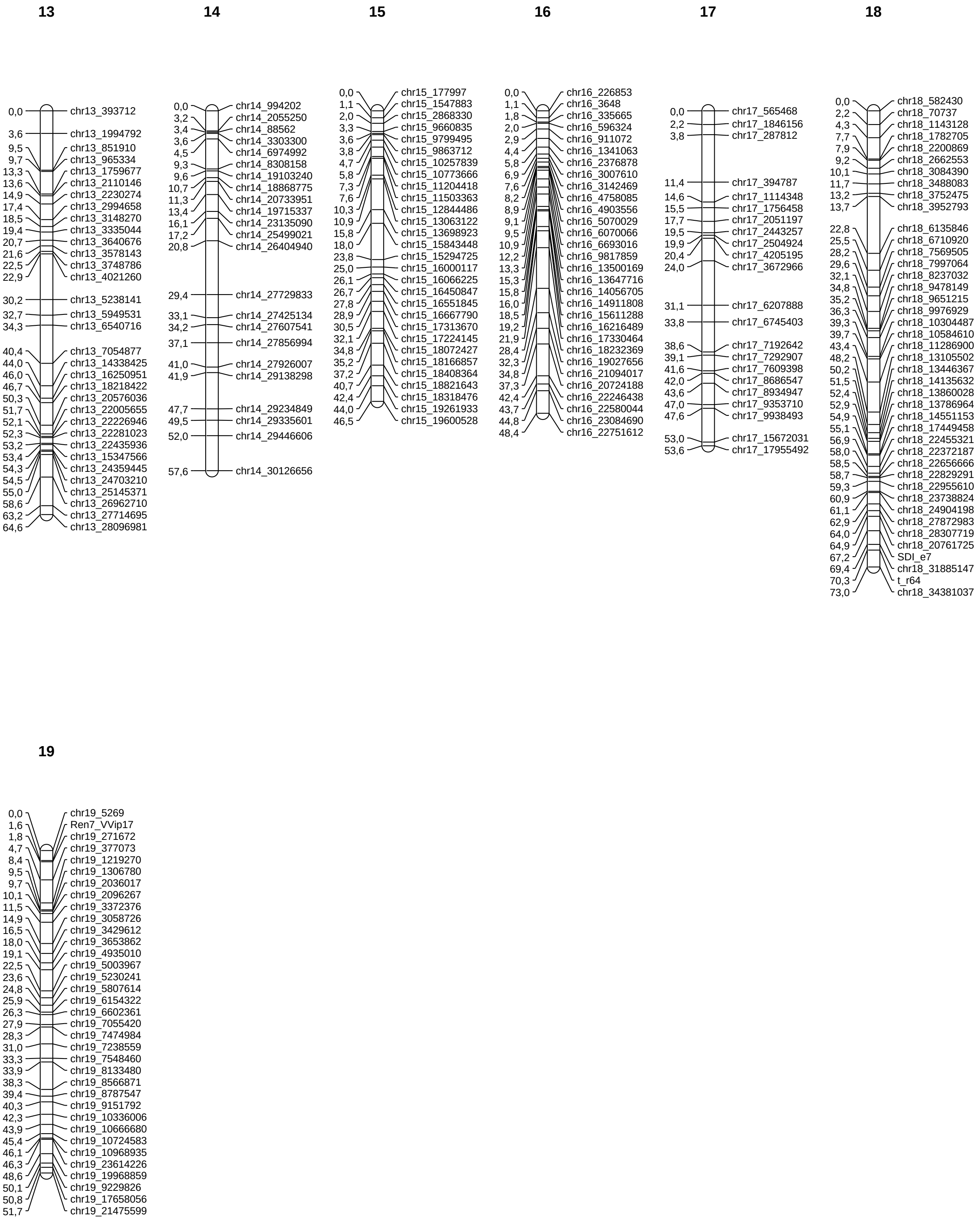

Supplementary Figure S2. Genetic linkage maps of the 19 linkage groups (LGs) of the 'Morio Muskat' × COxGT2 population Gf.2018-063, constructed with rhAmpSeq markers. Numbers on the left indicate genetic positions in centimorgans (cM), and marker names with their physical positions (Mb) are shown on the right according to the PN40024 reference genome (12X.v2).

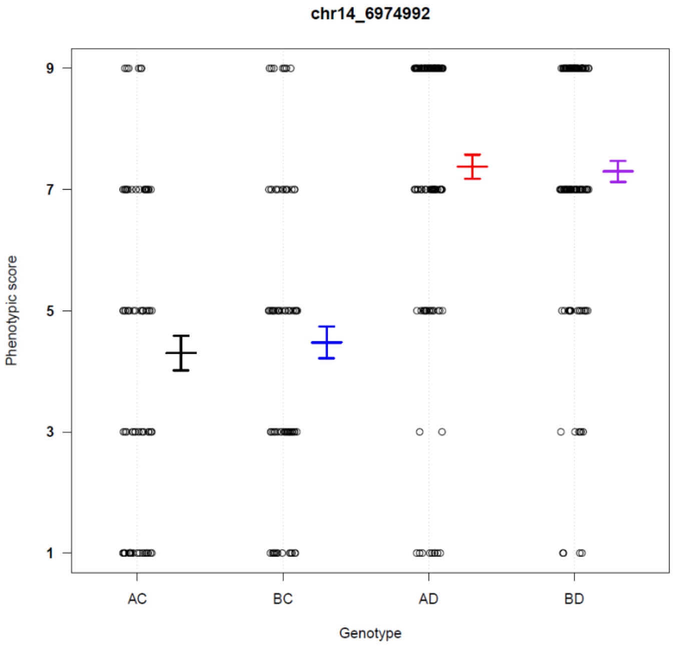

Supplementary Figure S3. Genotype classes (AC, BC, AD, BD) represent allele combinations of LODmax marker chr14_6974992 derived from the biparental cross ('Morio Muskat' × COxGT2; AB × CD), where A/B denote alleles from 'Morio Muskat' and C/D denote alleles from COxGT2. Phenotypic values correspond to downy mildew resistance scores based on the reversed OIV 452-1 scale, where score 1 indicates high resistance and score 9 indicates high susceptibility.

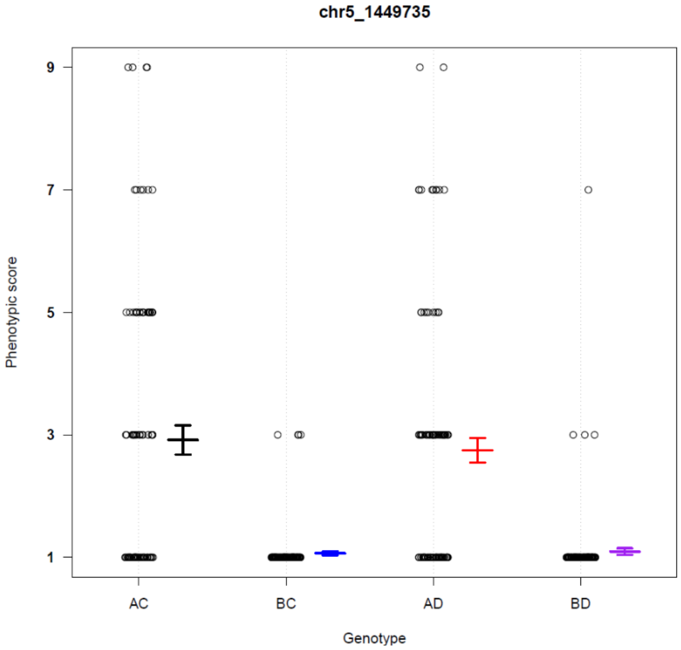

Supplementary Figure S4. Genotype classes (AC, BC, AD, BD) represent allele combinations of LODmax marker chr5_1449735 derived from the biparental cross ('Morio Muskat' × COxGT2; AB × CD), where A/B denote alleles from 'Morio Muskat' and C/D denote alleles from COxGT2. Phenotypic values correspond to leaf hair density based on the OIV 084 descriptor, where score 1 indicates absence or very low hair density and score 9 indicates very high (extremely dense) leaf hair.
